# A New Integrated GDL with Wavy Channel and Tunneled Rib for High Power Density PEMFC at Low Back Pressure and Wide Humidity

**DOI:** 10.1002/advs.202302928

**Published:** 2023-08-04

**Authors:** Can He, Qinglin Wen, Fandi Ning, Min Shen, Lei He, Yali Li, Bin Tian, Saifei Pan, Xiong Dan, Wei Li, Pengpeng Xu, Yiyang Liu, Zhi Chai, Yihuang Zhang, Wenming Liu, Xiaochun Zhou

**Affiliations:** ^1^ School of Nano‐Tech and Nano‐Bionics University of Science and Technology of China Hefei 230026 China; ^2^ Division of Advanced Nanomaterials Suzhou Institute of Nano‐tech and Nano‐bionics Chinese Academy of Sciences (CAS) Suzhou 215123 China; ^3^ WeiFu High‐technology Group Co., LTD. Wuxi 214000 China; ^4^ Key Laboratory of Precision and Intelligent Chemistry University of Science and Technology of China Hefei Anhui 230026 China

**Keywords:** integrated gas diffusion layers, low back pressure, micro‐tunnel ribs, proton exchange membrane fuel cells, wave channels

## Abstract

Proton exchange membrane fuel cells (PEMFCs) have garnered significant attention due to their high efficiency and low emissions. However, PEMFC always suffers mass transfer and water management in performance improvement. Herein, an integrated gas diffusion layer (GDL) with wavy channel and micro‐tunneled rib is designed and prepared to achieve faster and gentler mass transfer and excellent water management capability by laser engraving. Outstandingly, the new integrated GDL can use the back pressure of air as low as 0 and 50 kPa to respectively achieve 80% and 90% of fuel cell performance realized under pure oxygen. Such high performance is mainly due to the turbulent flow caused by wavy channel and express removing pathway of liquid water provided by micro‐tunneled rib. Moreover, the new integrated GDL also shows wide humidity tolerance from 40% to 100% and a very high specific volume power density of 16,300 W L^−1^ due to the thin thickness of new integrated GDL. This new integrated GDL is expected to be widely used in PEMFC and other energy conversion devices.

## Graphical abstract

An integrated gas diffusion layer (integrated GDL) with wavy channels and micro‐tunnel ribs is prepared by laser engraving. The integrated GDL exhibits excellent fuel cell performance under both back pressure and wide humidity range due to its special structure. This advantage can greatly reduce the energy consumption of the fuel cell system and make the fuel cell more widely used.

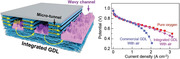

## 1 Introduction

PEMFC has been gaining significant attention due to its remarkable efficiency and low emissions. Power density, as indicated by the unit area, volume, or weight, is a crucial parameter for PEMFC.^[^ ^1^, ^2^, ^3^ ^]^ Unfortunately, poor mass transfer and water management in the cathode severely restrict improvements in power density. Especially at high current density, oxygen concentration polarization arises as the cathode rapidly consumes the air flow oxygen.^[^ ^4^, ^5^ ^]^ Meanwhile, the excess water generated at the cathode will accumulate in gas diffusion layer (GDL) and flow channel, ultimately obstructing gas transfer pathways and aggravating oxygen concentration polarization. Hence, the power density of PEMFC drops sharply.^[^ ^6^, ^7^ ^]^ To solve the above problems, there are three main approaches.

The first approach is the pure oxygen method. Compared with air supply, pure oxygen can effectively suppress the oxygen concentration polarization at high current density.^[^ ^8^ ^]^ The power density per unit area of fuel cells under pure oxygen supply can be 1.6‐3 times higher than that under air supply.^[^ ^9^, ^10^ ^]^ However, the production of pure oxygen is associated with extra costs and special devices. In addition, the flooding of fuel cells is still serious under pure oxygen supply at high current density due to the poor water management of GDL and flow fields. Therefore, this method is not widely adopted.

The second approach is the increasing back pressure method. Higher back pressure of the reactant gas can not only increase the concentration of oxygen but also reinforce the airflow, thus increasing oxygen transfer and water drainage. Currently, this method is the primary way to significantly improve the performance of fuel cell systems.^[^ ^11^, ^12^ ^]^ However, the higher back pressure of air requires a more powerful air compressor, which usually consumes ≈20% of total energy of the fuel cell system.^[^ ^13^, ^14^, ^15^ ^]^ Such high energy consumption of air compressors is a significant drawback for the future application of fuel cells. Hence, reducing the back pressure of air is highly desirable to lower down the power of the air compressor and improve the overall energy efficiency of fuel cell system.

**Figure 1:**
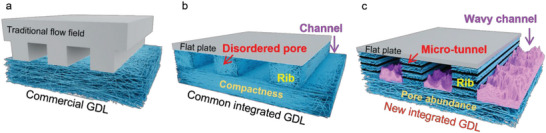
Schematic diagram of conventional flow field integrated GDL and our new integrated GDL. a) Commercial GDL and traditional flow fields. b) Common integrated GDL with flow field. c) Our new integrated GDL with wavy flow channel and porous rib with rich micro‐tunnels.

The third method is the flow field optimization method. Many studies have found that the mass transfer and water management of a fuel cell can be improved by changing the flow field structure in the bipolar plate, as shown in **Figure** **1**a.^[^ ^16^, ^17^, ^18^, ^19^, ^20^, ^21^, ^22^, ^23^, ^24^, ^25^, ^26^ ^]^ For example, multi‐serpentine flow fields,^[^ ^27^ ^]^ parallel flow fields,^[^ ^28^ ^]^ cross‐type flow fields^[^ ^29^ ^]^ and various bionic flow fields have been designed and prepared.^[^ ^30^, ^31^, ^32^, ^33^, ^34^ ^]^ To further enhance the water management capability, some researchers have tried porous flow fields such as metal foam^[^ ^35^, ^36^ ^]^ or graphene foam.^[^ ^37^, ^38^ ^]^ However, the porous flow field is so dense and unorganized that a huge pressure loss is generated, resulting in serious water residue inside the porous flow field.^[^ ^9^ ^]^ Moreover, an integrated GDL with a structure that integrates flow field and GDL has shown good performance in enhancing the gas transfer performance and water management capabilities of fuel cells in Figure 1b.^[^ ^9^, ^25^, ^26^, ^35^, ^39^ ^]^ However, This integrated GDL still has some disadvantages. First, the bottom of the common integrated GDL flow channel is relatively dense. Second, the pores in the ribs are disordered. Third, the common integrated GDL flow channel is a uniform flow channel without an ups and downs plane. These three aspects lead to difficult gas transport and water drainage at high current densities. Moreover, the preparation method, structure, and performance are yet to be fully explored, although the integrated GDL has shown attractive potential.

Ideally, it is desired to use low back pressure of air to achieve the highest power density realized under pure oxygen. In this work, we prepared a new integrated GDL (i‐GDL) with both GDL and flow field by a simple and easy process of laser engraving. The bottom of the new integrated GDL flow channels has a rich porous structure, and the new integrated GDL has wavy channels and porous ribs with rich micro‐tunnels, which provide excellent gas transfer and water management capabilities (Figure 1c). Remarkably, the new integrated GDL can use the back pressure of air as low as 0 kPa and 50 kPa, respectively, to achieve 80% and 90% of fuel cell performance obtained under pure oxygen. Moreover, the new integrated GDL also shows wide humidity tolerance from 40% to 100% and a very high specific volumetric power density of 16,300 W L^−1^ due to the thin thickness of new integrated GDL.

## 2 Results and Discussion

### 2.1 Preparation and Characterization of Integrated GDL

**Figure 2:**
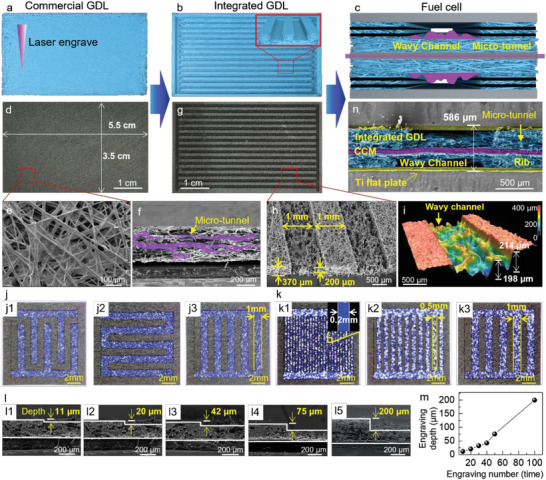
Preparation of new integrated GDL and assembly of fuel cell. a) Commercial gas diffusion layer (Commercial GDL) under laser engraving. b) Scheme of integrated GDL. c) Fuel cell made by the integrated GDL. (d) Optical picture of commercial GDL. e) Surface scanning electron eicroscope (SEM) image of commercial GDL. f) Cross‐section SEM image of commercial GDL. g) Optical picture of integrated GDL. h) SEM image of integrated GDL. i) 3D image measurement of integrated GDL. j) Optical picture of integrated GDL with different flow field structures. k) Optical picture of integrated GDL with different flow channel widths. l) SEM image of integrated GDL with different flow channel depths. m) The relationship between the number of etchings and the depth of etching. n) Cross‐section SEM image of a fuel cell made by integrated GDL.

**Figure** **2**a–c shows the methodology to prepare the new integrated GDL by a simple and easy process of laser engraving using commercial substrate, i.e., carbon paper, and to use it in fuel cells. Figure 2a shows that the surface of carbon paper was engraved by the laser marking machine. After laser engraving, flow channels can be obtained on carbon paper as shown in Figure 2b, and the cross‐section of integrated GDL is shown in the inset of Figure 2b. Subsequently, the integrated GDL is used to assemble fuel cell in Figure 2c, which shows that the new integrated GDL has wavy channels and porous ribs with layered tunnels. The wavy channel is generated by laser engraving, while the porous rib with layered tunnel is generated by the layer‐by‐layer stack of carbon fiber in the commercial carbon paper substrate.

Figure 2d shows the carbon paper with dimensions 5.5 cm in length and 3.5 cm in width. The surface of carbon paper is flat and smooth before laser engraving. The SEM image in Figure 2e,f shows that the surface of carbon paper is composed of long carbon fibers, and has a highly porous structure. The cross‐section of carbon paper in Figure 2f shows that the carbon paper is organized by the layer‐by‐layer stack of carbon fiber. This structure provides rich layered tunnels in the plane of carbon paper, and also in the ribs of the new integrated GDL.

After laser engraving, the integrated GDL with a very well‐ordered flow channel structure was prepared, as shown in Figure 2g. Then, the prepared integrated GDL was treated with PTFE solution to enable good hydrophobicity (contact angle is 141.9° in Figure S1, Supporting Information). In this integrated GDL, the width of both flow channel and rib is 1 mm, and the depth of flow channel is 200 µm (Figure 2h). The SEM image in Figure 2h and the 3D profile scan in Figure 2i and Figures S2 and S3 (Supporting Information) also show that the unengraved location retains the flat surface, while the engraved location exhibits a wavy channel. The maximum depth reaches 214 µm. The flat surface is beneficial to the contact between GDL and bipolar plate, and also to reducing the contact resistance. The wavy channel is promising to bring the advantage of mass transfer in the new integrated GDL. In addition, the integrated GDL prepared by laser engraving still maintains a high tensile strength compared with the unengraved GDL, as shown in Figure S4 (Supporting Information). Although the laser destroys the carbon fiber structure on the surface, the bottom of the integrated GDL still maintains a complete carbon fiber structure, as shown in Figure S5 (Supporting Information). Therefore, the integrated GDL exhibits good stability.

The laser engraving method in this work has many advantages, including fast rate, easy design, flexible dimension, and low cost to modify the flow field parameters such as flow channel type, width, and depth. The flow fields with interdigitated, serpentine, snake, and parallel flow channels can be easily prepared (Figure 2j and Figure S6, Supporting Information). The flow channel width can be precisely adjusted from 0.1 mm to 2 mm as shown in Figure 2k and Figure S6 (Supporting Information). In addition, the depth of the integrated GDL channel can also be adjusted from 11 µm to 200 µm by laser engraving (Figure 2l,m and Figure S6, Supporting Information). After careful characterization, the integrated GDL with parallel flow field is the best one due to the lower pressure drop only 3.63 kPa at 1000 mL min^−1^ (Figure S7, Supporting Information), which will be mainly used in following sections.

The new integrated GDL is assembled in a single fuel cell (Figure 2n and Figure S8, Supporting Information). As shown in Figure 2n, the single fuel cell is composed of a catalyst‐coated membrane (CCM), two pieces of new integrated GDL, and two slides of Ti flat plate. The channel and rib structures in the fuel cell are easily identifiable. Remarkably, this fuel cell structure assembled with integrated GDL does not require traditional bipolar plates with flow channels but only needs a flat, thin metal or graphite plate as a collector plate. Compared to traditional bipolar plates, the flat type of plate significantly reduces thickness and production costs. The total thickness of the single fuel cell is only 586 µm, which is highly conducive to reducing the volume of fuel cell stack and achieving high specific volume power density.

### 2.2 High Performance and Low Energy Consumption of Integrated GDL under Low Back Pressure

**Figure 3:**
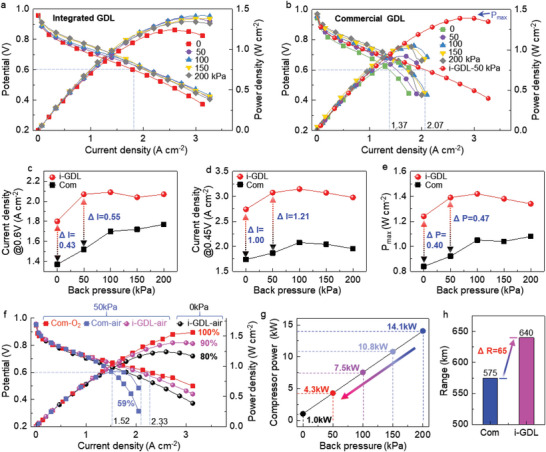
Fuel cell performances of integrated and commercial GDLs with different back pressure. a) Polarization curves of integrated GDL under different back pressure. b) Polarization curves of commercial GDL. c) Comparing the current densities at 0.6 V of integrated and commercial GDLs. d) Comparing the current densities at 0.45 V of integrated and commercial GDLs. e) Comparing the peak power density of integrated and commercial GDLs. f) Performance of integrated and commercial GDLs under hydrogen‐air and hydrogen‐oxygen conditions. g) Estimation of power consumption of air compressors under different back pressure. h) Estimation of driving range of fuel cell cars using the integrated and commercial GDLs.

The polarization curves of different rib widths show that the fuel cell performance is the highest when the rib area to flow channel area ratio is 1, as shown in Figure S9 (Supporting Information).^[^ ^40^, ^41^ ^]^ In order to further determine whether the new integrated GDL can achieve high performance under low back pressure, the back pressure of supplied air varies from 0 to 200 kPa at the low stoichiometric ratio of air only 1.5. Remarkably, **Figure** **3**a shows that, even at 0 kPa back pressure, the polarization curve with the new integrated GDL doesn't exhibit obvious oxygen concentration polarization at the high current density larger than 3 A cm^−2^, indicating high mass transfer of new integrated GDL. The fuel cell performance slightly increases when the back pressure increases from 0 kPa to 50 kPa. Interestingly, the integrated GDL maintains a relatively high and very close fuel cell performance, when the back pressure increases from 50 kPa to 200 kPa. All polarization curves in Figure 3a show that the integrated GDL has a good mass transfer effect under low back pressures.

For comparison, the performance of commercial GDL with conventional flow channels was also tested. Figure 3b shows that the commercial GDL performs poorly at a back pressure of 0 kPa, which encounters the concentration polarization at the current density as low as 1.37 A cm^−2^. With the increase of back pressure, although the polarization curve of commercial GDL improves obviously, it still has a very obvious concentration polarization at low current density. The reason is that the liquid water generated by the reaction was not removed in time and flooded in the commercial GDL. In contrast, the red dot curve of integrated GDL in Figure 3b shows much higher performance. Therefore, the integrated GDL has much better mass transfer than the commercial GDL, and the water in the catalytic layer can be quickly removed at high current density.

To compare the performance of integrated and commercial GDL in detail, three performance parameters, including the current density at 0.6 V, the current density at 0.45 V, and the peak power density, are plotted versus back pressure, as shown in Figure 3c–e, respectively. Figure 3c shows that the current density at 0.6 V of integrated GDL is always much higher than that of commercial GDL. Even at 0 kPa back pressure, the current density at 0.6 V of integrated GDL can reach 1.8 A cm^−2^, which is much higher than that of commercial GDL 1.37 A cm^−2^. Remarkably, Figure 3c also shows that the difference in current density at 0.6 V reaches the maximum value of 0.55 A cm^−2^ at 50 kPa back pressure. In addition, comparing the electrochemical impedance spectroscopy (EIS) of integrated GDL and the commercial GDL at 0.6 V in Figure S10 (Supporting Information), the integrated GDL has a smaller mass transfer resistance than commercial GDL.

Figure 3d shows that, even at 0 kPa back pressure, the current density of integrated GDL is 2.74 A cm^−2^, while the current density of commercial GDL is only 1.74 A cm^−2^. The current density difference of the two GDLs can reach as high as 1.00 A cm^−2^ at 0 kPa back pressure. When the back pressure increases from 0 kPa to 50 kPa, the current density of integrated GDL at 0.45 V is significantly improved to ≈3 A cm^−2^, which is 1.5 times of commercial GDL at 200 kPa. However, the current density slightly decreases when the back pressure exceeds 100 kPa, indicating the new integrated GDL is better to work at lower back pressure.

Figure 3e shows that the peak power density of integrated GDL is 1.24 W cm^−2^ at the back pressure of 0 kPa, while the peak power density of commercial GDL is only 0.84 W cm^−2^. Moreover, the peak power densities of integrated GDL increase to 1.39 W cm^−2^ and 1.42 W cm^−2^ at the back pressure of 50 kPa and 100 kPa, respectively. Remarkably, the peak power density of integrated GDL at 50 kPa can also reach 1.3 times the commercial GDL at 200 kPa. The fuel cell performance of the integrated GDL was compared to that of the commercial GDL under the same flow field type and flow field parameters. The results show that the fuel cell performance of the integrated GDL is 1.8 times higher than that of the commercial GDL at 0 kPa, as shown in Figure S11 (Supporting Information).

Due to the special structure of integrated GDL, it can achieve high current density without back pressure. With low back pressure, excess water can be easily removed through gas transfer. In addition, the gas diffusion speed from the GDL to the catalyst layer is accelerated, resulting in increased oxygen content in the catalyst layer and improved fuel cell performance. However, when the back pressure exceeds 100 kPa, it becomes difficult to drain the excess water from the fuel cell. This can hinder gas diffusion and reduce fuel cell performance.^[^ ^11^, ^12^, ^42^, ^43^, ^44^, ^45^ ^]^ Therefore, Figure 3c–e shows a similar trend, indicating that the integrated GDL has high mass transfer at low back pressure.

To compare the performance of integrated GDL in pure oxygen and air, the fuel cell performance under air and pure oxygen conditions was compared at the low back pressure of 0 kPa and 50 kPa. The fuel cell performance of commercial and integrated GDLs is almost the same (≈1.5 W cm^−2^) under pure oxygen conditions (Figure S12, Supporting Information). Figure 3f shows that the peak power density of commercial GDL in air conditions is only 59% of that in pure oxygen conditions. Surprisingly, Figure 3f also shows that the new integrated GDL can achieve 80% and 90% of high fuel cell performance realized under pure oxygen when using air with a back pressure of 0 and 50 kPa, respectively. Therefore, this experiment proves that the mass transfer ability of integrated GDL is so high that oxygen and water transfer in it at low back‐pressure air can reach the standard of pure oxygen.

The extremely high mass transfer ability of new integrated GDL has a huge advantage in improving the energy efficiency of the whole fuel cell system. In a fuel cell system, the air compressor is a high‐energy cost part, which costs ≈20% energy of total system. The reduction of air back pressure can significantly reduce the power consumption of air compressor as shown in Figure 3g and Figure S13 (Supporting Information) improve the output power of fuel cell, and improve the energy efficiency of total fuel cell system. Currently, the high air back pressure of 200 kPa is usually used with the commercial GDL to obtain a good performance. At such high pressure, the power consumption of air compressor is as high as 14.1 kW in Figure 3g and SI‐13.^[^ ^13^, ^14^, ^15^ ^]^ Outstandingly, if the new integrated GDL has even better fuel cell performance at the low air back pressure of 50 kPa, the power consumption of air compressor is only 4.3 kW, which is only≈1/3 of commercial GDL. If the air back pressure is reduced to 0 kPa, the power consumption of air compressor can be even as low as 1.0 kW, which is only 1/14 of commercial GDL. Therefore, the use of a new integrated GDL can save a lot of energy, and also reduce the cost of air compressors due to the lower pressure requirement.

The saved energy through using the new integrated GDL can greatly improve the driving range of fuel cell cars. We calculate the driving range of fuel cell cars by replacing the commercial GDL with the new integrated GDL according to the publicly reported data (Figure S14 and Supporting Information 14, Supporting Information).^[^ ^15^, ^46^ ^]^ Figure 3h shows that the driving range can be increased from 575 km to 640 km by using the new integrated GDL, which is a 65 km improvement compared to commercial GDL. Therefore, the integrated GDL has high potential in saving enough energy to obviously improve the driving range of fuel cell cars, so that the fuel cell has a wider range of application scenarios.

### 2.3 Outstanding Water Management Capability of Integrated GDL

**Figure 4:**
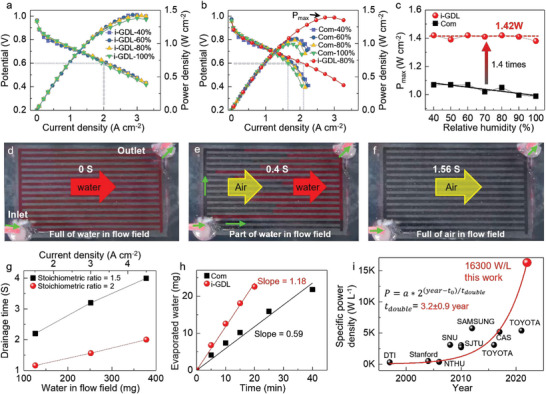
Water management capability of integrated GDL (i‐GDL) and commercial GDL with different humidity. a) Polarization curves of integrated GDL with different humidity. b) Polarization curves of commercial GDL with different humidity. c) Peak power density versus humidity for both integrated GDL and commercial GDL. d) The integrated GDL is filled with water before gas purging. e) The integrated GDL is filled with partial water during gas purge. f) The integrated GDL is filled with air after gas purging. g) Gas purging time of integrated GDL at different water volume and air stoichiometric ratios. h) Water evaporation rate of integrated GDL and traditional flow field. i) Volume‐specific power density of PEMFC reported in the literature.

In order to know the humidity tolerance ability of the new integrated GDL, the fuel cell performance of integrated GDL is further studied at a wide humidity range. Interestingly, **Figure** **4**a exhibits that the integrated GDL always shows similar high performance (≈1.5 W cm^−2^) at wide humidity from 40% to 100% (Figure S15, Supporting Information), for more polarization curves). The fuel cell with the integrated GDL shows neither over‐drying at low humidity 40% nor flooding at high humidity 100%. Therefore, the new integrated GDL shows wide humidity tolerance.

As a comparison, Figure 4b and Figure S15 (Supporting Information) show the fuel cell performance of commercial GDL at different humidity. The commercial GDL starts to show severe concentration polarization from the current density of ≈1 A cm^−2^ even at low humidity of 40%, because a large amount of generated water covers the reaction sites of catalytic layer and blocks the gas transport path from the diffusion layer to the catalytic layer.^[^ ^47^, ^48^, ^49^ ^]^ Moreover, the mass transfer becomes even worse when the humidity increases to 100%. However, the integrated GDL has no obvious concentration polarization in the polarization curve at an even high current density of 3 A cm^−2^ (red dot curve in Figure 4b). Notably, Figure 4c shows that the peak power density of integrated GDL shows wide humidity tolerance from 40% to 100%, while that of commercial GDL keeps decreasing with the increase of humidity. The peak power density of integrated GDL is 1.4 times that of commercial GDL. Therefore, the integrated GDL has a much wider and higher humidity tolerance than the commercial GDL.

Water generated in the cathode catalyst layer exits in two forms: gaseous water and condensed water (liquid water). Since the flow field channel in the integrated GDL is only 200 µm deep, it is necessary to determine whether liquid water can be efficiently removed from the channel. To test this, the integrated GDL's ability to remove liquid water was evaluated, as shown in Figure 4d–f. A certain amount of water (252 mg water, produced at current density of 3 A cm^−2^ in 1 min, calculated in Figure S16, Supporting Information) was injected into the flow field channel of integrated GDL and then was purged out by airflow (the stoichiometric ratio of air is 2). Figure 4d–f shows that it takes only 1.56 seconds to completely purge out all the water (drainage time) from the flow channel. Figure 4g and Figures S17 and S18 (Supporting Information) further show the drainage time at different air stoichiometric ratios and different amounts of water, i.e., different current densities. Higher air stoichiometric ratio can greatly shorten the drainage time, indicating faster drainage ability. The drainage time is always < 2 s when the stoichiometric ratio is only 2. Figure 4g also shows that the drainage time gradually increases with increasing water volume when the stoichiometric ratio of air is constant. Even if the water in flow field is close to 400 mg (generated at 4.5 A cm^−2^ for 1 min), the drainage time is only 4 seconds (black dot curve in Figure 4g).

In addition, the water evaporation rate across the integrated GDL and commercial GDL is also compared, as shown in Figure 4h and Figures S19 and S20 (Supporting Information). When the stoichiometric ratio of air is 1.5, the water evaporation rate of integrated GDL is 1.18 mg/(min cm^2^), which is much faster than that of a traditional flow field of 0.59 mg/(min cm^2^). At other air stoichiometric ratios, the integrated GDL also shows a faster water evaporation rate (Figure S20, Supporting Information). Therefore, the integrated GDL has great water management capabilities in removing both liquid water and gaseous water.

Due to the high performance (≈1.5 W cm^−2^) and thin thickness (586 µm), the fuel cell with the integrated GDL is easy to achieve high volume‐specific power density. As shown in Figure 2j, the total thickness of a single fuel cell is only 586 µm, which is much thinner than that of traditional fuel cells 1‐2 mm. The main reason for the reduction of thickness is due to the thin thickness of integrated GDL containing both GDL and flow field, which occupies >80% of thickness in traditional fuel cells. The fuel cell assembled with the integrated GDL has a much smaller volume and much higher volume‐specific power density of 16,300 W L^−1^, which is much higher than that in the literature (Figure 4i and Table S1).^[^ ^50^, ^51^, ^52^, ^53^, ^54^, ^55^, ^56^, ^57^, ^58^ ^]^ The volume‐specific power density of fuel cells can be fitted by the exponential function below and doubles every *t*_double_ = 3.6 ± 0.6 years.

(1) $$P=a*2^{\left(year-t_{0}\right)/t_{\mathrm{double}}}$$

### 2.4 Excellent Fuel Cell Performance Stability

**Figure 5:**
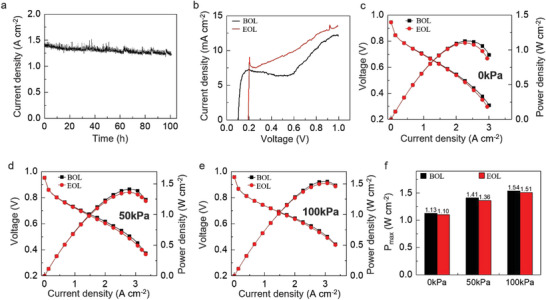
The fuel cell long‐time stability test. a) Stability test for 100 h at 0.6 V. b) hydrogen crossover comparison of the beginning‐of‐life (BOL) and the end‐of‐life (EOL). c) Polarization curves of integrated GDL of the BOL and the EOL at 0 kPa. d) Polarization curves of integrated GDL of the BOL and the EOL at 50 kPa. e) Polarization curves of integrated GDL of the BOL and the EOL at 100 kPa. f) Comparing the peak power density of integrated GDL of the BOL and the EOL.

This work also studies the impact of the integrated GDL on the fuel cell performance during long‐term discharge. As shown in **Figure** **5**a, the constant voltage discharge process of the integrated GDL for 100 h was recorded. The results show that the integrated GDL current density is very stable during constant voltage discharge for 100 h. After 100 h of constant voltage discharge, the current density of the integrated GDL is only slightly attenuated. In order to further assess whether the integrated GDL long‐term test has pierced the proton exchange membrane, we recorded the hydrogen crossover before and after the long‐term test, as shown in Figure 5b. Before the long‐term test, the hydrogen crossover of the integrated GDL is 6.5 mA at 0.4 V. Following 100 h of constant voltage discharge, the hydrogen crossover of the integrated GDL is 8.5 mA at 0.4 V. This indicates that even after prolonged constant voltage discharge, the integrated GDL does not cause PEM puncture or high hydrogen crossover.

In addition, the fuel cell performance before and after 100 h of constant voltage discharge were compared as shown in Figure 5c–f. The results show that under the back pressure of 0 kPa, the peak power density is 1.13 W cm^−2^ before constant voltage discharge, and the peak power density is 1.10 W cm^−2^ after constant voltage discharge for 100 h, the fuel cell performance only drops by 30 mW cm^−2^. When the back pressure is 50 kPa, before the constant voltage discharge, the peak power density is 1.41 W cm^−2^, and after 100 h of constant voltage discharge, the peak power density is 1.36 W cm^−2^, the fuel cell performance only drops by 50 mW cm^−2^, as shown in Figure 5d,f. When the back pressure is 100 kPa, before and after long‐term constant pressure discharge, the fuel cell performance drops by 30 mW cm^−2^, as shown in Figure 5e,f. These results show that the fuel cells assembled with integrated GDL have excellent stability.

### 2.5 Discussion of Mass Transfer Mechanism of Integrated GDL and Commercial GDL

The above research shows that the integrated GDL has much higher mass transfer, water management, and consequently higher fuel cell performance than the commercial GDL. This is due to the advanced structure of integrated GDL, which significantly affect mass transfer and water management. In addition, The fuel cell performance of the TGP‐060 (the thickness of TGP‐060 is almost the same as the bottom thickness of integrated GDL) and the integrated GDL was compared under the same conditions, as shown in Figure S21 (Supporting Information). The results show that the integrated GDL has good mass transfer ability and excellent fuel cell performance. In this section, we will compare the structures of integrated GDL and commercial GDL with traditional flow fields in detail, and discover the source of high mass transfer structure.

**Figure 6:**
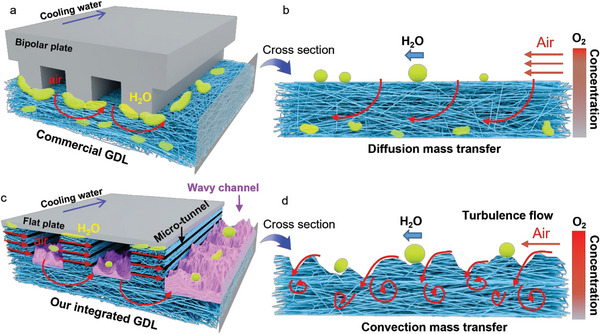
Mass transfer mechanism of integrated GDL and commercial GDL. a) Front view of commercial GDL mass transfer. b) Side view of commercial GDL mass transfer. c) Front view of integrated GDL mass transfer. d) Side view of integrated GDL mass transfer.

**Figure** **6**a shows that the commercial GDL and the traditional flow field are separated from each other. This structure has two shortcomings that strongly and negatively affect mass transfer. First, water generated in the catalyst layer easily condenses into liquid water at the interface of rib and carbon paper (yellow blocks in Figure 6a) and hinders the mass transfer blow to the rib. Since the temperature on the interface of rib and carbon paper is relatively lower than in other locations due to the cooling water,^[^ ^59^, ^60^ ^]^ liquid water is easy to be generated there.^[^ ^35^ ^]^ Hence, thicker thickness of carbon paper substrate can contain more liquid water under the rib and is conducive to mass transfer at high current density. Second, the mass transfer between flow field channel and catalyst layer is usually through slow diffusion as shown in Figure 6b. Because of flat surface of commercial GDL in the flow field channel as shown in Figure 6b and the low Reynolds number (SI‐21, Supporting Information), the flow in the channel usually follows a transition flow state between laminar flow and turbulent flow. Under the transition flow state, the mass transfer between the flow field channel and the catalyst layer is usually through slow diffusion perpendicular to flow direction, as shown in Figure 6b. Hence, thinner thickness of carbon paper substrate is conducive to the mass transfer through diffusion in channel at high current density. Therefore, it is better to use carbon paper with a thicker thickness under rib and a thinner thickness in channel. However, the thickness of current carbon paper is homogenous, and the mass transfer of the traditional structure with separated commercial GDL and flow field is strongly limited by slow diffusion and condensed water.

To solve above problems, we have designed a new integrated GDL with both wavy channels and tunneled ribs, as shown in Figure 6c. In this design, the bipolar plate is just a thin and flat plate. This structure has two advantages that strongly and positively affect mass transfer. First, the tunneled porous rib can quickly remove the condensed liquid water at the interface of rib and bipolar plate (yellow blocks in Figure 6c), because the micro‐tunnels in the rib can provide a highly expressive pathway for gas flow. Second, wider oxygen convection because of wavy surface of the new integrated GDL in the flow field channel, as shown in Figure 6d, and the high Reynolds number of the fluid in the integrated GDL flow channel (SI‐22, Supporting Information), the flow state in the channel follows turbulent flow,^[^ ^61^ ^]^ which cause fast mass transfer. The gas transfer in the flow channel generates a very large number of vortices in Figure 6d, which constantly change the direction of gas flow and thus facilitates the gas and water transfer in the integrated GDL. Due to the characterization of vortices, the mass exchange between channel and catalyst layer is very fast and gentle. In contrast, transition flow often causes the airflow to purge a certain part of MEA, resulting in continuous drying or wetting of the MEA. Based on the above analysis, the integrated GDL exhibit significantly higher performance than commercial GDL due to its faster and gentler mass transfer and water management capabilities.

## 3 Conclusion

In summary, a new type of integrated GDL with GDL and flow field has been successfully designed and prepared by laser engraving. This method can accurately, rapidly, and easily control key parameters such as channel width and type, thereby offering a fresh perspective on the development of integrated GDLs. The new integrated GDL has wavy channels and porous ribs with rich layered micro‐tunnels, which can provide excellent gas transfer and water management capabilities. Remarkably, the new integrated GDL has the capability to achieve 80% and 90% of fuel cell performance realized under pure oxygen at back pressure as low as 0 and 50 kPa, respectively. In comparison with commercial GDL, the performance of new integrated GDL at 0 kPa back pressure is higher than commercial GDL at 200 kPa. This huge advantage can greatly improve the energy efficiency of the whole fuel cell system since the air compressor consumes a large amount of energy. As a result, it is estimated that the driving range of fuel cell cars can have a 65 km improvement by simply replacing the commercial GDL with the new integrated GDL. Moreover, the new integrated GDL also shows wide humidity tolerance from 40% to 100%. Remarkably, this fuel cell structure, assembled with integrated GDL, does not require traditional bipolar plates with flow channels but only needs a flat and very thin metal or graphite plate as a collector plate. This structure can greatly reduce the total thickness of the single fuel cell to only 586 µm, which is conducive to achieving a very high specific volume power density of 16,300 W L^−1^. Therefore, the new integrated GDL can realize the ideal of using low back pressure of air to achieve the same power density as realized under pure oxygen. Due to the innovation of wavy channels and micro‐tunneled ribs, this new integrated GDL is also expected to be widely used in PEMFC and other energy conversion devices.

## 4 Experimental Section

### Materials

All chemicals were obtained commercially and used without further purification unless otherwise stated. Polytetrafluoroethylene (PTFE, Sigma‐Aldrich Co., Ltd.). The GDL used to prepare the integrated GDL was obtained from Toray, the GDL was TGP‐120.^[^ ^62^ ^]^ The integrated GDLs were treated with PTFE to achieve hydrophobicity, and the integrated GDLs had 5% PTFE content. This work used the commercial GDL SGL 28BC, and the SGL 28BC was obtained from Sigracet. The commercial GDL (SGL 28BC) had 5% PTFE content and a thickness of 230 µm.^[^ ^63^ ^]^

### Flow Field Information

In this work, unless otherwise specified, the integrated GDL utilized a parallel flow field with a depth of 200 µm, and both the flow field width and rib width were 1 mm. The traditional flow field was a three‐snake flow field with a flow channel depth of 1 mm, and both the flow field width and rib width were 1 mm.

### Preparation of Integrated GDLs

UG software was used to design the flow channel, and then imported the designed flow channel structure into the laser marking machine, The focal length of laser marking machine was adjusted to 10.4 cm, the current was set to 5 A, and the number of etchings times was 40 times. The GDL was placed on the adsorption platform for laser etching to prepare an integrated GDLs

### Hydrophobic Treatment of Integrated GDLs

Integrated GDLs were treated with PTFE to achieve hydrophobicity, with a degree of 5%. First, the commercial PTFE was diluted to prepare a solution with a mass fraction of 3%. Second, immersing the prepared integrated GDLs in the PTFE solution for 20 s, drying them at 100 °C, and weighing the mass, until the desired mass fraction was achieved.

### Preparation of MEAs

For the hydrogen‐air and hydrogen‐oxygen test, the membrane electrode assembly (MEA) was prepared as follows: an integrated GDL‐based fuel cell was assembled, with TGP‐120‐integrated GDL as the cathode and TGP‐090‐integrated GDL as the anode. Catalyst‐coated membrane (CCM) used in this study was obtained from Suzhou Sinero Technology Co., Ltd (China). The proton exchange membrane (PEM) of CCM was Gore M820.15 (15 µm). The catalyst of this CCM was JM Hispec 9100 (55.5–58.5%), and the anode and cathode Pt loadings were 0.24 and 0.48 mg cm^−2^, respectively.

### Physical Characterizations of Integrated GDL

The morphology and structure of integrated GDLs were characterized by scanning electron microscope (Quanta 250 FEG, S‐4800). The shape and size of integrated GDLs were recorded by a camera, and the contact angle image of integrated GDLs was recorded by a contact angle measuring device (SINDIN SDC‐200s) with a high‐speed camera of Dahang. The integrated GDL was prepared by Dazu UV laser marking machine (EP‐15‐THG). The 3D scanned images of integrated GDL and commercial GDL were characterized by a Laser profile measuring instrument (Keyence, VK‐k1000).

### The Test of PEMFC

The performance of PEMFCs was tested with Toyo Fuel Cell Test System equipped with 890e Fuel Cell Test Loads (Scribner Associates Inc.) and 885‐HS Fuel Cell Potentiostat (Scribner Associates Inc.). PEMFCs polarization curves and electrochemical impedance spectroscopy (EIS) under different back pressures were recorded at 80 °C of fuel cell temperature and 80% of humidity, and H_2_ of anode and the air of cathode flow rate were 0.5 and 2.0 NL min^−1^, respectively.

### Polarization Curves

PEMFCs polarization curves under different humidity were recorded at 80 °C of fuel cell temperature and 100 kPa back pressures. The flow rate of H_2_ at anode and the flow rate of air at cathode were 0.5 and 2.0 NL min^−1^, respectively.

PEMFCs polarization curves under H_2_‐O_2_ conditions were recorded at 80 °C of fuel cell temperature, 80% of humidity, and 100 kPa back pressures. The flow rate of H_2_ at anode and the flow rate of air at cathode were 0.3 and 0.3 NL min^−1^, respectively.

### Electrochemical Impedance Spectroscopy (EIS) Test

The test conditions of EIS were the same as those of polarization curve. The EIS was tested at the sweep frequency mode at the potential of 0.6 V. The frequency range of EIS test was 10 kHz–0.1 Hz. The amplitude of AC signal was kept at 10% of DC current.

### Liquid Water Removing Test

First, water production was calculated under different current densities. The corresponding quality of water was then injected into the flow channel. A fixed flow of air was introduced into the flow channel, and the time from the introduction of air to the complete emptying of water was recorded.

### Gaseous Water Removing Test

First, prepared an integrated GDL of 0.6 × 5 cm and a filter paper of same size. Added 25 mg of water onto the filter paper, placed the filter paper under the integrated GDL, and assembled and weighed the mass. Passed the corresponding airflow through the system, and recorded the mass every 5–10 min until the mass no longer changed. The traditional flow field gaseous water removing test was consistent with the integrated GDL.

## Conflict of Interest

The authors declare no conflict of interest.

## Supporting information

Supporting Information

## Data Availability

The data that support the findings of this study are available from the corresponding author upon reasonable request.
