# Supplementary material for: A New Integrated GDL with Wavy Channel and Tunneled Rib for High Power Density PEMFC at Low Back Pressure and Wide Humidity
Source: Adv Sci (Weinh). 2023 Aug 4;10(28):2302928. doi: 10.1002/advs.202302928 (PMC10558662; doi:10.1002/advs.202302928)
Supplement: Supplementary file 1 — Supporting Information [file ADVS-10-2302928-s001.pdf]

ADVANCED SCIENCE
Open Access

Supporting Information

for *Adv. Sci.*, DOI 10.1002/advs.202302928

A New Integrated GDL with Wavy Channel and Tunneled Rib for High Power Density PEMFC at Low Back Pressure and Wide Humidity

*Can He, Qinglin Wen, Fandi Ning, Min Shen, Lei He, Yali Li, Bin Tian, Saifei Pan, Xiong Dan, Wei Li, Pengpeng Xu, Yiyang Liu, Zhi Chai, Yihuang Zhang, Wenming Liu and Xiaochun Zhou**

Supplementary Information

A New Integrated GDL with Wavy Channel and Tunneled Rib for High Power Density PEMFC at Low Back Pressure and Wide Humidity

*Can He, Qinglin Wen, Fandi Ning, Min Shen, Lei He, Yali Li, Bin Tian, Saifei Pan, Xiong Dan, Wei Li, Pengpeng Xu, Yiyang Liu, Zhi Chai, Yihuang Zhang, Wenming Liu, Xiaochun Zhou****

Can He, Qinglin Wen, Yali Li, Bin Tian, Saifei Pan, Xiong Dan, Zhi Chai, Xiaochun Zhou

School of Nano-Tech and Nano-Bionics, University of Science and Technology of China, Hefei, 230026, China

E-mail: xczhou2013@sinano.ac.cn

Can He, Qinglin Wen, Fandi Ning, Min Shen, Lei He, Yali Li, Bin Tian, Saifei Pan, Xiong Dan, Wei Li, Pengpeng Xu, Yiyang Liu, Zhi Chai, Xiaochun Zhou

Division of Advanced Nanomaterials, Suzhou Institute of Nano-tech and Nano-bionics, Chinese Academy of Sciences (CAS), Suzhou, 215123, China

Yihuang Zhang, Wenming Liu

WeiFu High-technology Group Co., LTD. Wuxi, 214000, China

Xiaochun Zhou

Key Laboratory of Precision and Intelligent Chemistry, University of Science and Technology of China, Hefei, Anhui 230026, China.

Content

SI-1 Integrated GDL Hydrophobicity....3

SI-2 The SEM image of integrated GDL....4

SI-3 3D image of commercial GDL and integrated GDL....5

SI-4 Tensile strength of integrated GDL....6

SI-5 The bottom SEM image of integrated GDL....7

SI-6 Parameter regulation of the integrated GDL....8

SI-7 Pressure drop of the integrated GDL....10

SI-8 Integrated GDL fuel cell assembly process....12

SI-9 Performance of integrated GDL with different ribs....13

SI-10 Performance of integrated GDL and commercial GDL with different back pressure....15

SI-11 Performance under the same configuration of commercial GDL and integrated GDL....18

SI-12 Performance of integrated GDL and commercial GDL under pure oxygen conditions....20

SI-13 Relationship between air compressor power consumption and back pressure Compressor power....21

SI-14 Integrated GDL range prediction....22

SI-15 Performance of integrated GDLs with different humidity....23

SI-16 Fuel cells water production calculation....25

SI-17 The removal time of different water volumes (the stoichiometric ratio of air flow is 2)....26

SI-18 The removal time of different water volumes (the stoichiometric ratio of air flow is 1.5)....28

SI-19 Integrated GDL gaseous water testing device....30

SI-20 Comparison of gas water transfer for different GDLs....31

SI-21 Performance comparison of integrated GDL and TGP-060....33

SI-22 The flow form of gas in the flow channel....35

SI-23 Summary of volume specific power density of fuel cells literatures in recent year....36

SI-1 Integrated GDL Hydrophobicity

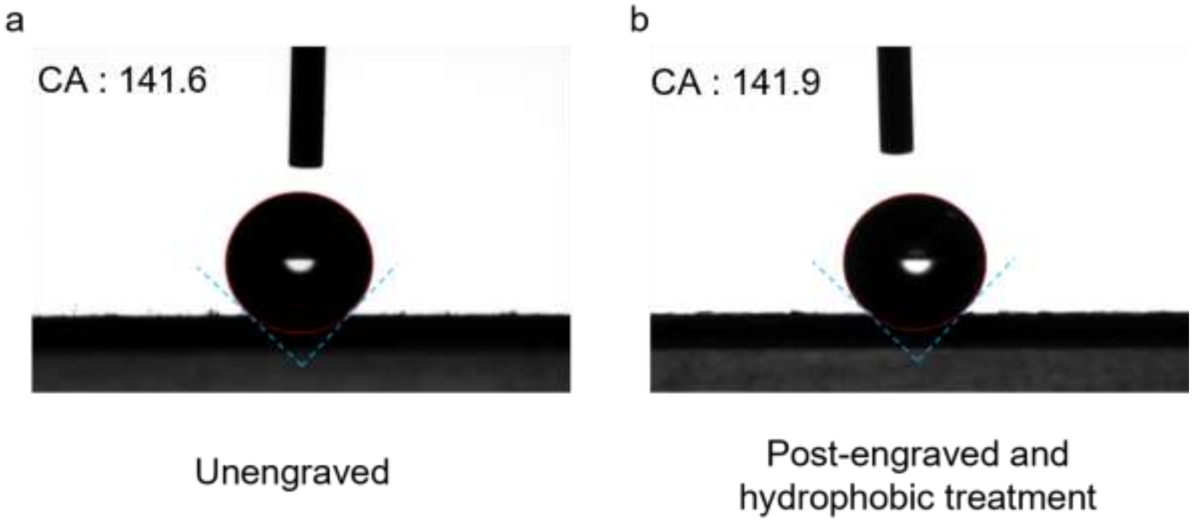

Figure S1. Water contact angles of GDL. (a) Water contact angle of un-engraved GDL. (b) Water contact angle of integrated GDL after hydrophobic treatment.

SI-2 The SEM image of integrated GDL

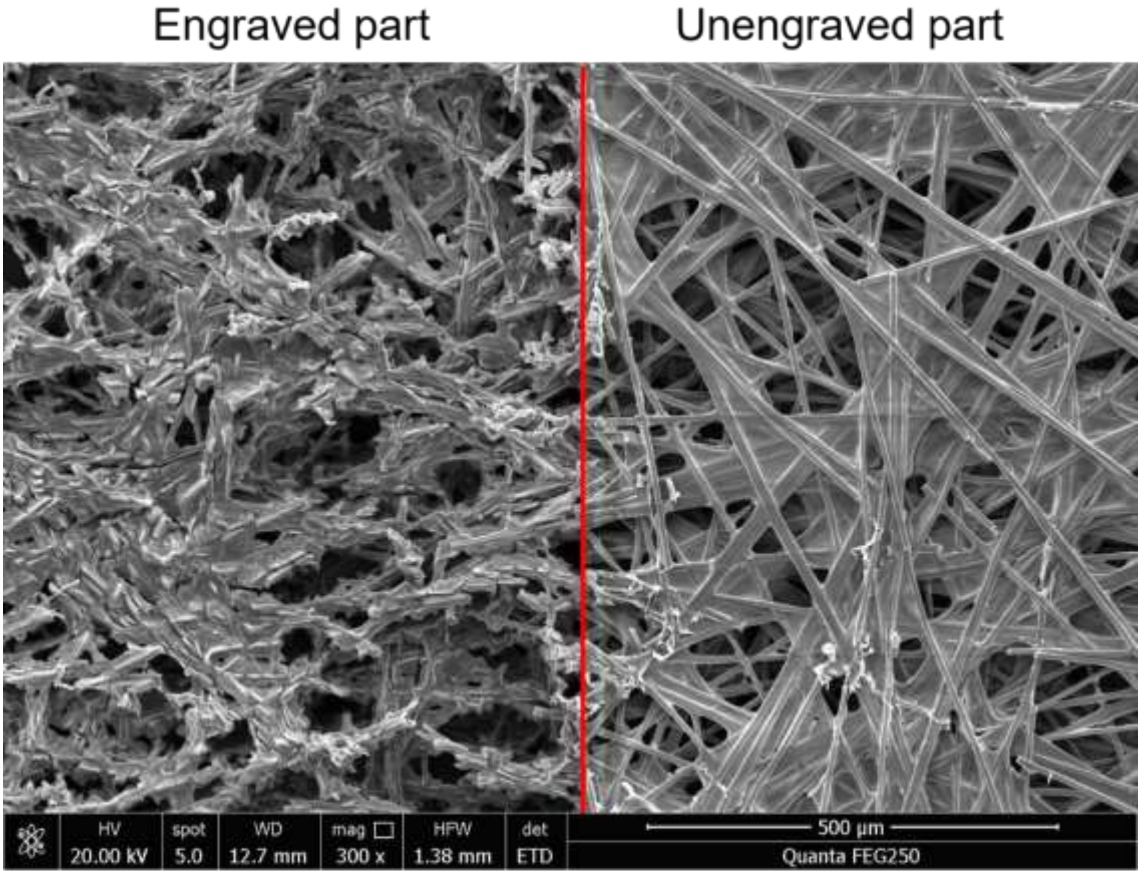

Figure S2. SEM image of GDL surface before and after engraving.

SI-3 3D image of commercial GDL and integrated GDL

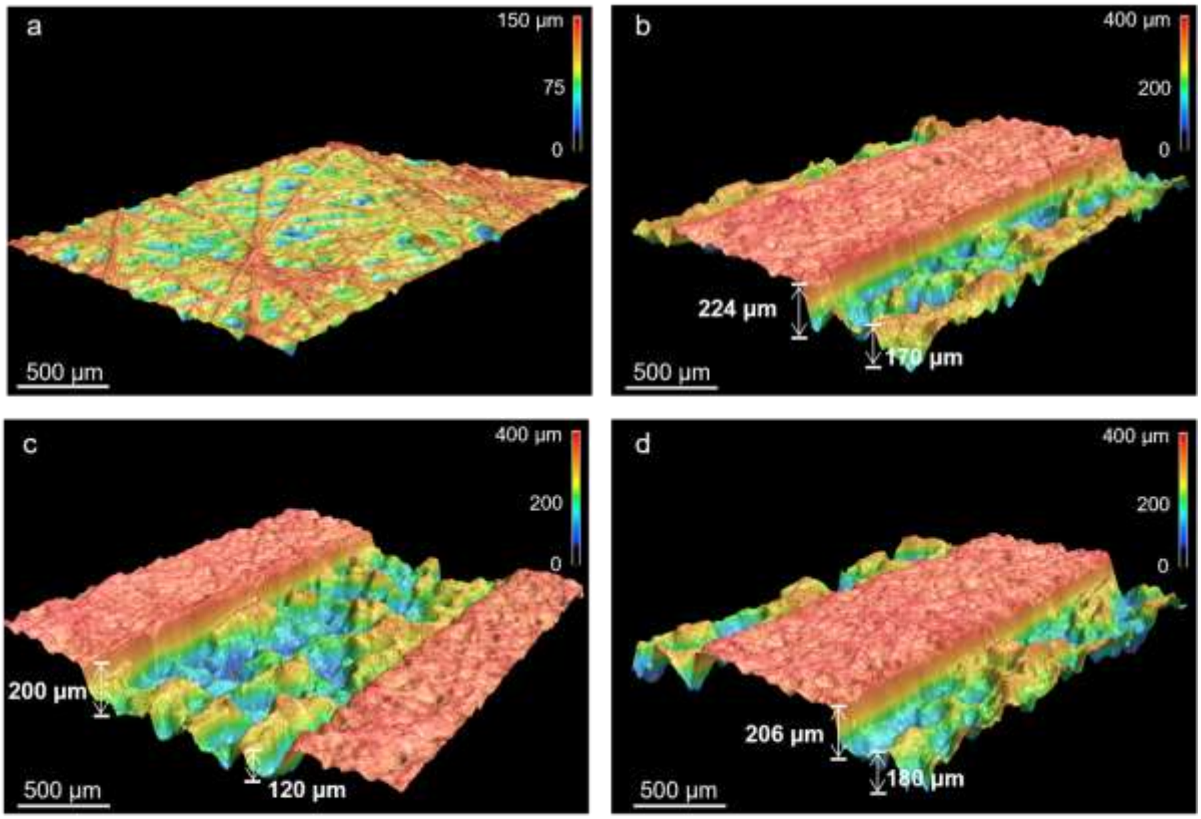

Figure S3. 3D image measurement of commercial GDL and integrated GDL. (a) 3D image measurement of commercial GDL. (b), (c), (d) 3D image measurement of integrated GDL.

As shown in the figure, **Figure S3**a shows the commercial GDL surface, the surface of commercial GDL has a rich porous structure. **Figure S3**b-d shows the integrated GDL surface, the red part area is the un-engraved location retains the flat surface, while the engraved location exhibits wavy channel.

SI-4 Tensile strength of integrated GDL

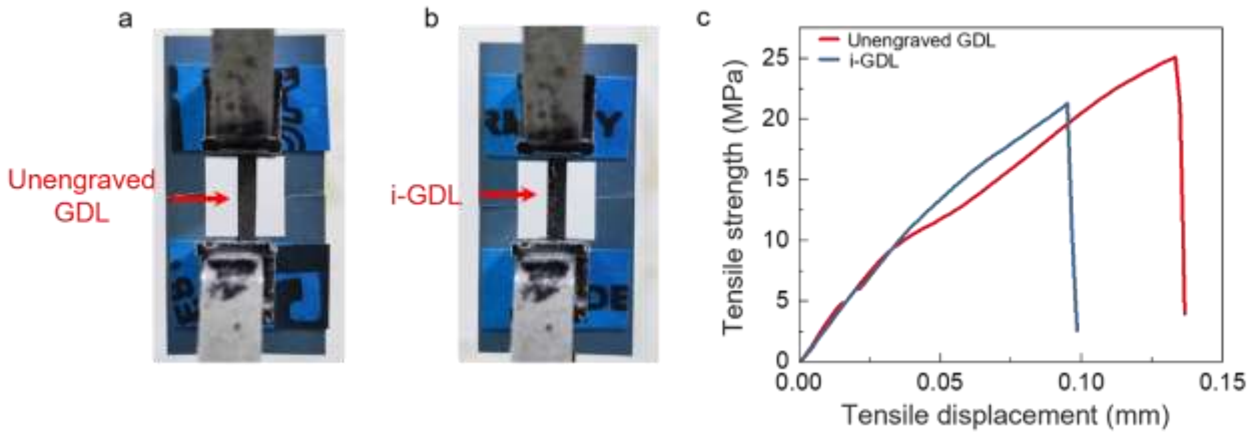

Figure S4. The tensile strength tests. (a) The optical image of unengraved GDL. (b) The optical image of integrated GDL. (c) The tensile curve of unengraved GDL and integrated GDL.

To compare the strength of integrated GDL and unengraved GDL, we performed tensile tests on unengraved GDL and integrated GDL under identical conditions, as shown in **Figure S4**.. The tensile strength of the unengraved GDL was 25.1 MPa, and the tensile strength of the integrated GDL was 21.3 MPa. Although the strength of integrated GDL is slightly lower than that of unengraved GDL, but it still maintains a high strength. This is because the integrated GDL prepared by laser engraving only etches part of the carbon fiber, and the structure of the bottom of the flow channel was not damaged.

SI-5 The bottom SEM image of integrated GDL

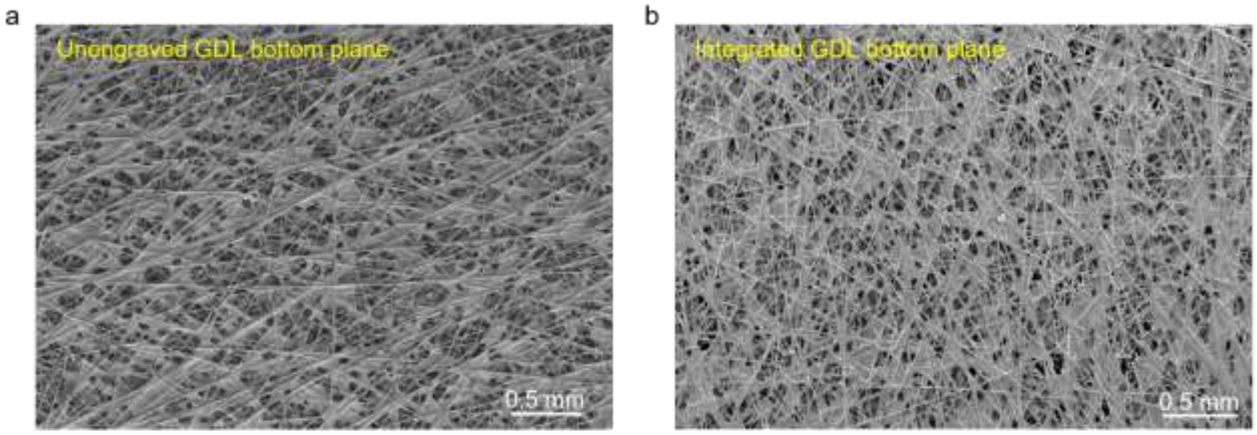

Figure S5. The SEM image of bottom plane. (a) The SEM image of unengraved GDL bottom plane. (b) The SEM image of integrated GDL bottom plane.

To further demonstrate the stability of the integrated GDL, we also carried out SEM characterization of the plane at the bottom of the flow channel, as shown in **Figure S5**. **Figure S5**a is the SEM image of the bottom of the unengraved GDL, and **Figure S5**b is the SEM image of the bottom of the integrated GDL. The results show that the bottom of the flow channel still maintains the original GDL structure, and the carbon fibers are hardly damaged. Therefore, the integrated GDL prepared by laser engraving still has strong stability.

SI-6 Parameter regulation of the integrated GDL

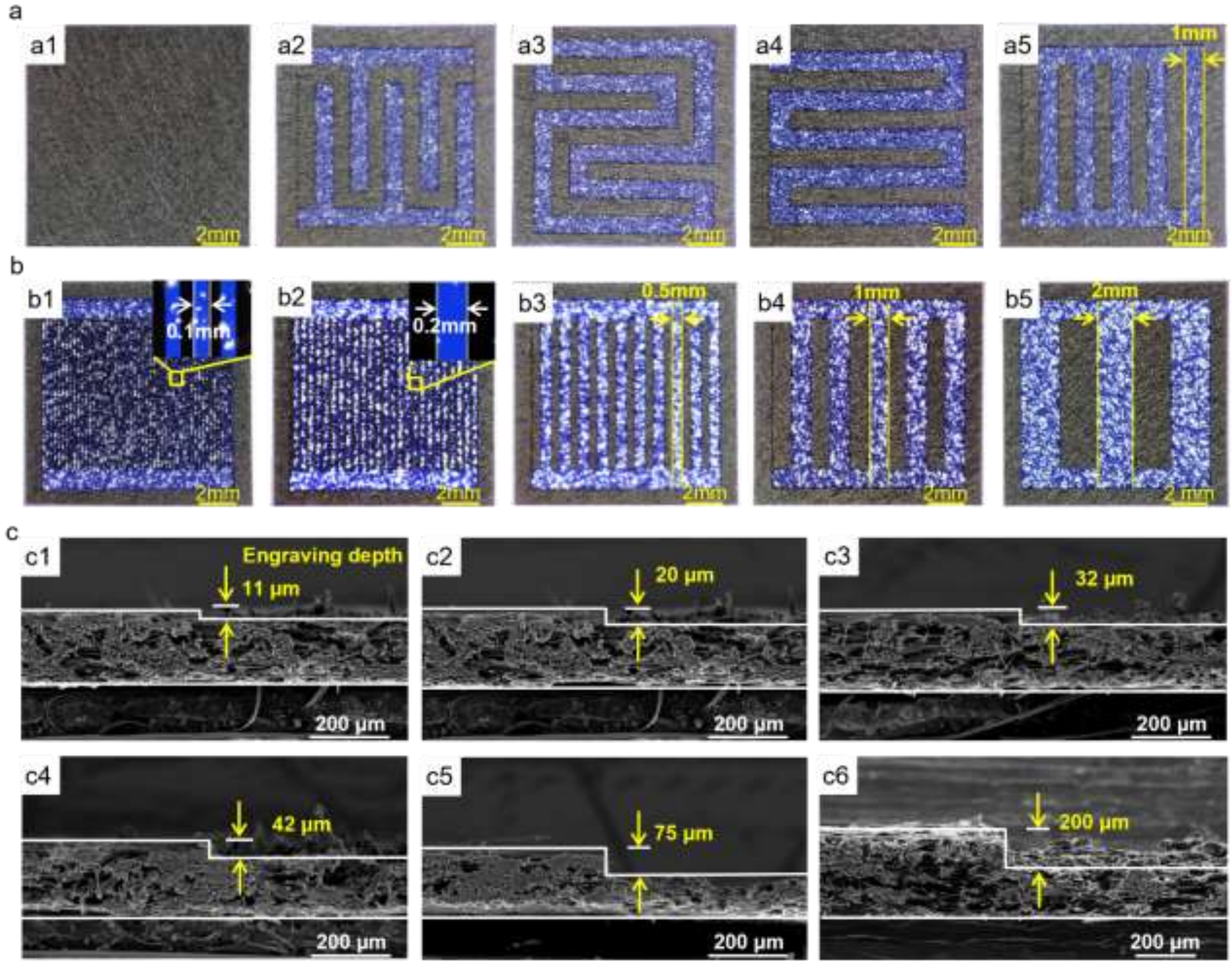

Figure S6. Preparation and pressure drop of the integrated GDL with different parameters. (a) Optical picture of commercial GDL (b) Optical picture of integrated GDL with Serpentine flow field. (c) Optical picture of integrated GDL with 0.1 mm flow channel widths. (d) Optical picture of integrated GDL with 2 mm flow channel widths.

The laser engraving method has the advantages of fast speed, simple design, flexible size, and low cost of flow field parameter adjustment. Various types of flow channels were fabricated on GDL by this method. **Figure S6**a shows the integrated GDL with different flow field types, including single serpentine channel, double snake channel, interdigitated channel, and parallel flow channel. Moreover, by this method we can precisely regulate the runner width from 0.1 mm to 2 mm, as shown in **Figure S6**b. The blue area in **Figure S6**b is the channels engraved by laser, while the black area is the raw location without engraved. Further, by adjusting the laser focal length and etching time, the etching depth can be adjusted, and this preparation method can obtain a flow field with a

wavy flow channel. Therefore, the laser engraving method here can conveniently and quickly fabricate any type of flow field, whose shape can be designed by software.

SI-7 Pressure drop of the integrated GDL

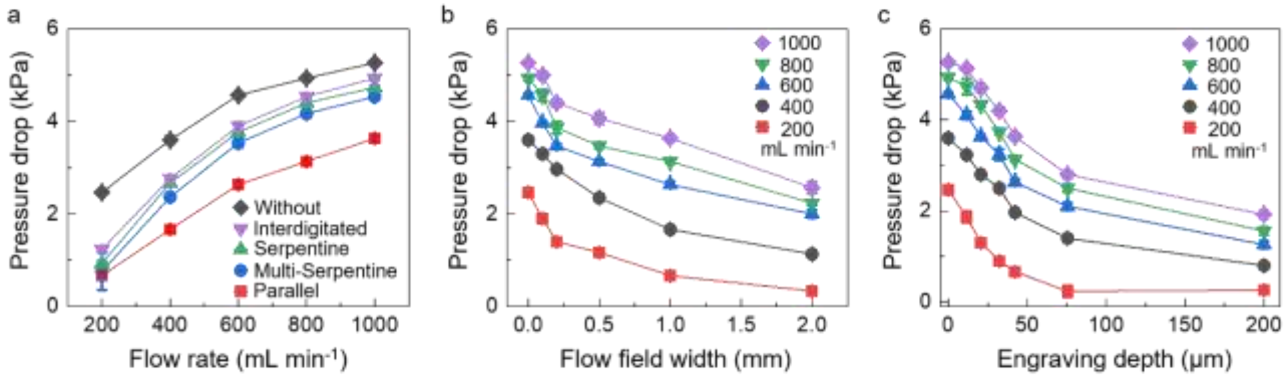

Figure S7. Pressure drop of the integrated GDL different flow rates. (a) Pressure drop with different types of flow field widths. (b) Pressure drop with different flow field widths. (c) Integrated GDL pressure drop with different engraving depths.

As the integrated GDL is very thin, pressure drop becomes an important characteristic of the flow field with different parameters. In order to find the integrated GDL with better flow field, the pressure drop of different types and flow field width and depth was characterized. **Figure S7**a shows the pressure drop of different types of integrated GDL. Under the same gas flow rate, the commercial GDL without flow channel has the large pressure drop. Differential pressure drop for GDL integrating different flow channel types, the order of pressure drop is interdigitated integrated GDL > single snake integrated GDL > double snake integrated GDL > parallel integrated GDL, with the continuous increase of air flow, the pressure drop is also increased gradually. However, the integrated parallel GDL has a lower pressure drop than the integrated GDL of other types flow channels and commercial GDL, and the pressure drop at 1000 mL min^{-1} is only 3.63 kPa. This also shows that the GDL with the integrated parallel flow channel has less resistance in the mass transfer process.

Further, the pressure drop test was carried out on the prepared integrated GDL with different channel widths. As the gas flow rate decreases, the pressure drop of the integrated GDL gradually decreases at the same flow path width as shown in **Figure S7**b. This is because the higher the gas

flow rate, the higher the resistance in the flow path, resulting in an integrated GDL with a larger pressure drop. However, under the same air flow rate, as the flow channel width increases, the pressure drop gradually decreases. Especially when the flow channel width increases from 0.1 mm to 1 mm, the pressure drop changes more sharply. When the flow channel width increases from 1mm to 2 mm, the pressure drop changes less. The main reason is that the smaller of the width of the flow channel, the greater the resistance of the air flow through the flow channel, the greater the pressure drop.

The pressure drop of the integrated GDL is also affected by the depth of the prepared flow channel. Therefore, the influence of the depth of the flow field of the pressure drop of the integrated GDL has also been studied. The pressure drop of the integrated GDL at different depths as shown in **Figure S7**c. The results show that under a certain gas flow rate, the pressure drop gradually decreases with the increase of the engraving depth. When the etching depth is greater than 75 μm, the pressure drop decreases slowly, which indicates that when the channel depth reaches a certain level, the pressure drop will gradually become stable. In summary, the preparation of integrated GDL by laser engraving have good universality, and it also precisely controls the integrated GDL.

SI-8 Integrated GDL fuel cell assembly process

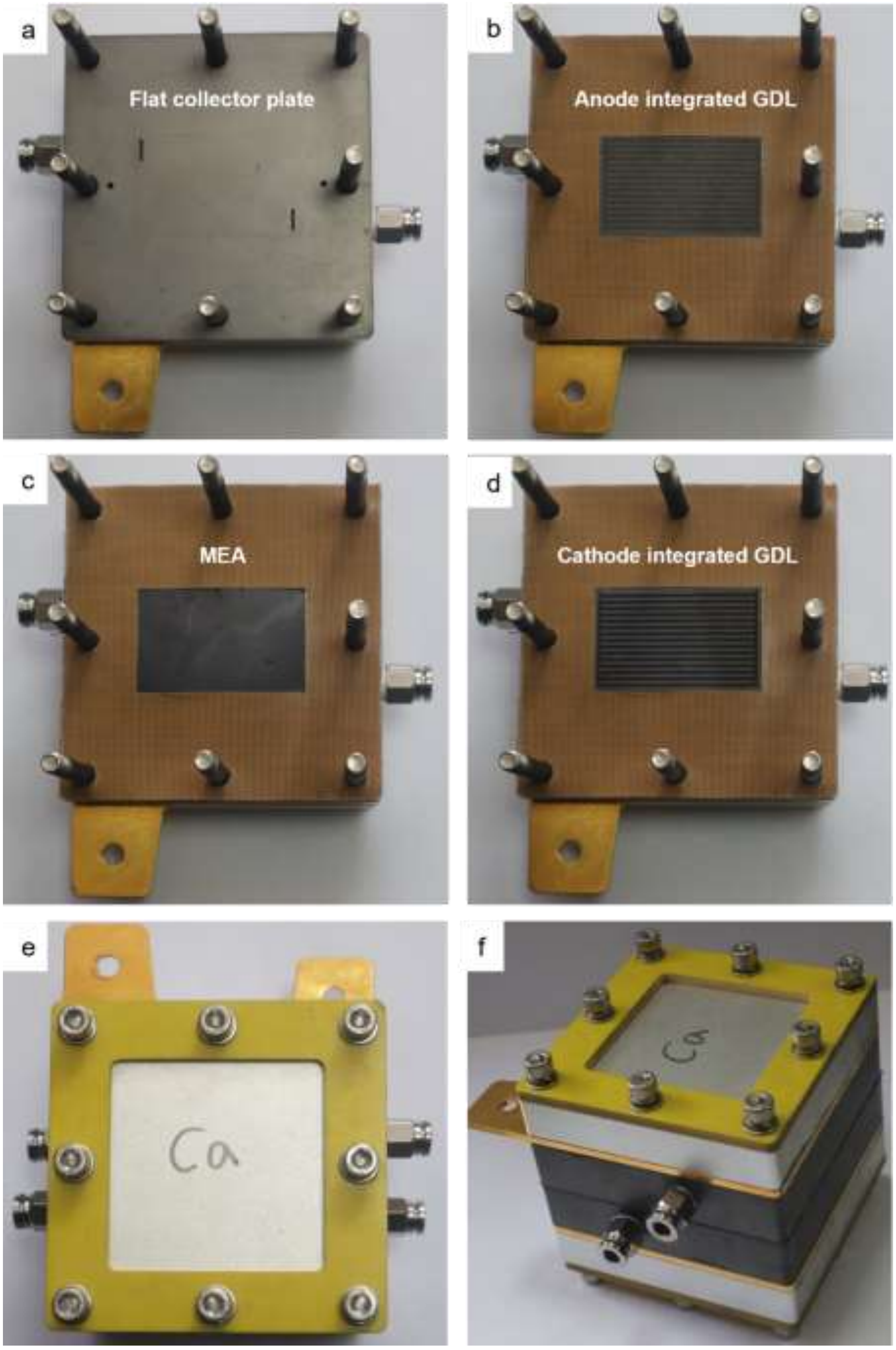

Figure S8. Integrated GDL fuel cell assembly demonstration. (a) Flat type collector plate. (b) Cathode integrated GDL. (c) MEA. (d) Anode integrated GDL. (e) Top view of fuel cell. (f) Side view of fuel cell.

Figure S8 shows the assembly process of the integrated GDL fuel cell. The structure of the fuel cell assembled with the integrated GDL is very simple, and the assembly the fuel cell is easier.

SI-9 Performance of integrated GDL with different ribs

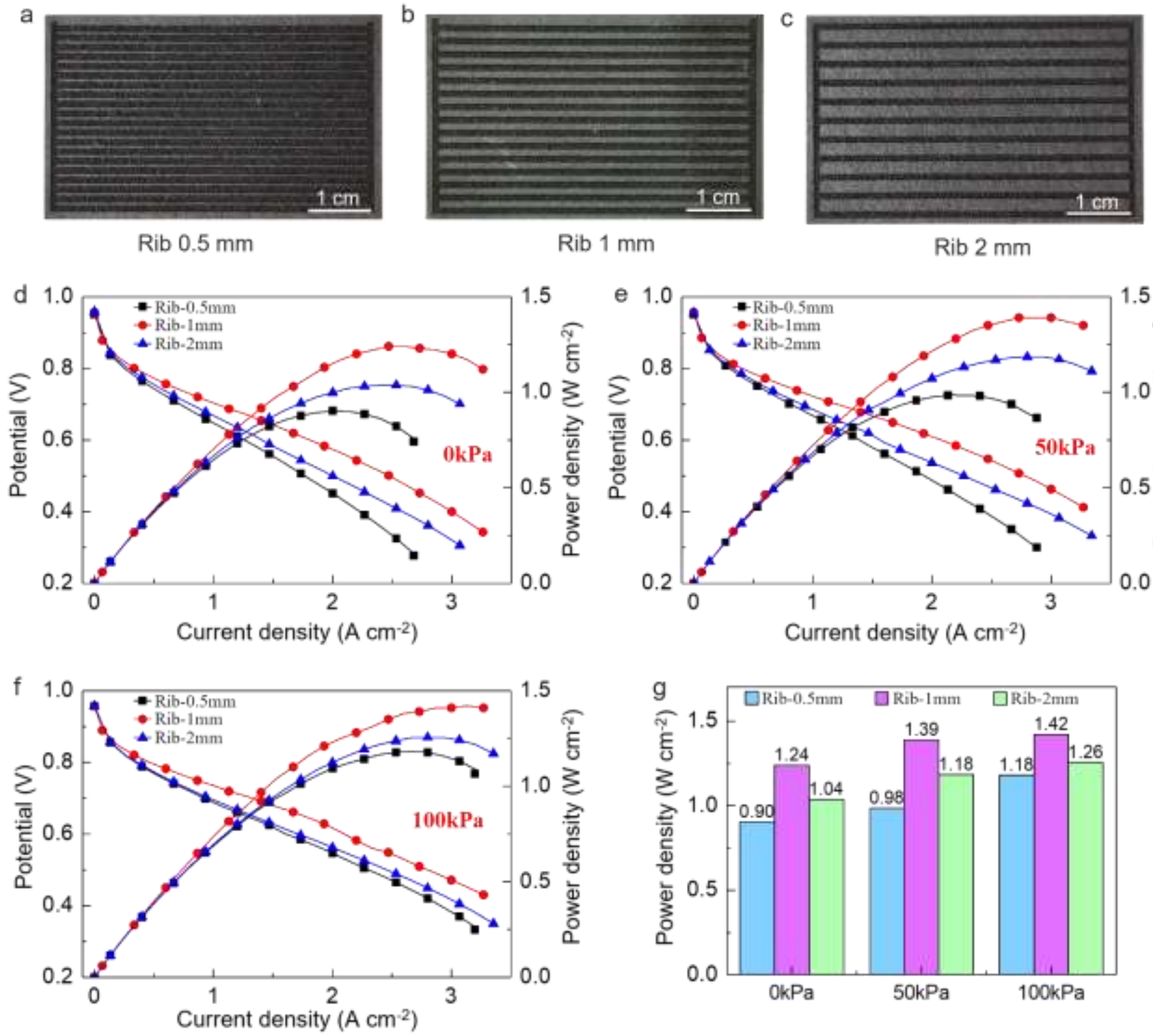

Figure S9. Comparison fuel cell performance of the integrated GDL with rib widths of 0.5 mm, 1 mm, and 2 mm. (a) Optical photograph of the integrated GDL with a rib widths of 0.5 mm.(b) Optical photograph of the integrated GDL with a rib widths of 1 mm. (c) Optical photograph of the integrated GDL with a rib widths of 2 mm. (d) Polarization curves of the integrated GDL with a rib widths of 0.5 mm, 1 mm, and 2 mm at 0 kPa. (e) Polarization curves of the integrated GDL with a rib widths of 0.5 mm, 1 mm, and 2 mm at 50 kPa. (f) Polarization curves of the integrated GDL with a rib widths of 0.5 mm, 1 mm, and 2 mm at 100 kPa. (g) Comparing the peak power density of the integrated GDL with a rib widths of 0.5 mm, 1 mm, and 2 mm under different backpressure.

In order to further discuss the effect of rib width on fuel cell performance, we prepared integrated GDL with rib widths of 0.5 mm, 1 mm, and 2 mm, as shown in **Figure S9**a-c, these three

integrated GDL have the same flow channel widths and depths. And the fuel cell performance of the fuel cell with rib widths of 0.5 mm, 1 mm, and 2 mm were compared under different back pressures, as shown in **Figure S9**d-g.

In the **Figure S9**d-f show that the integrated GDL with a rib width of 1 mm have excellent fuel cell performance. Under the same conditions, the performance of the integrated GDL fuel cell with the rib width of 0.5 mm and 2 mm is worse than that of the integrated GDL fuel cell with the rib width of 1 mm. Especially when the back pressure is 50 kPa, the integrated GDL fuel cell performance is 1.39 W cm^{-2} when the ridge width is 1mm, and the integrated GDL fuel cell performance is 0.98 W cm^{-2} and 1.18 W cm^{-2} when the ridge width is 0.5 mm and 2 mm, respectively.

The possible reasons are as follows. Under the same channel width, when the rib width is 0.5 mm, the contact resistance of the integrated GDL will become large. The rib is a porous structure, which will affect the distribution of reactant gas when the rib width is 0.5 mm, resulting in poor fuel cell performance. When the rib width is 2 mm, due to the reduction of the number of flow channels, the gas diffusion from the GDL to the catalytic layer becomes slower, which will also reduce the performance of the fuel cell. According to previous studies, channel and rib widths may affect the species diffusion, mass transfer, contact resistance and further affect fuel cell performance. The fuel cell performance is the best when the ratio of the channel area to the rib area is 1, which coincides with our test.[1, 2]

SI-10 Performance of integrated GDL and commercial GDL with different back pressure

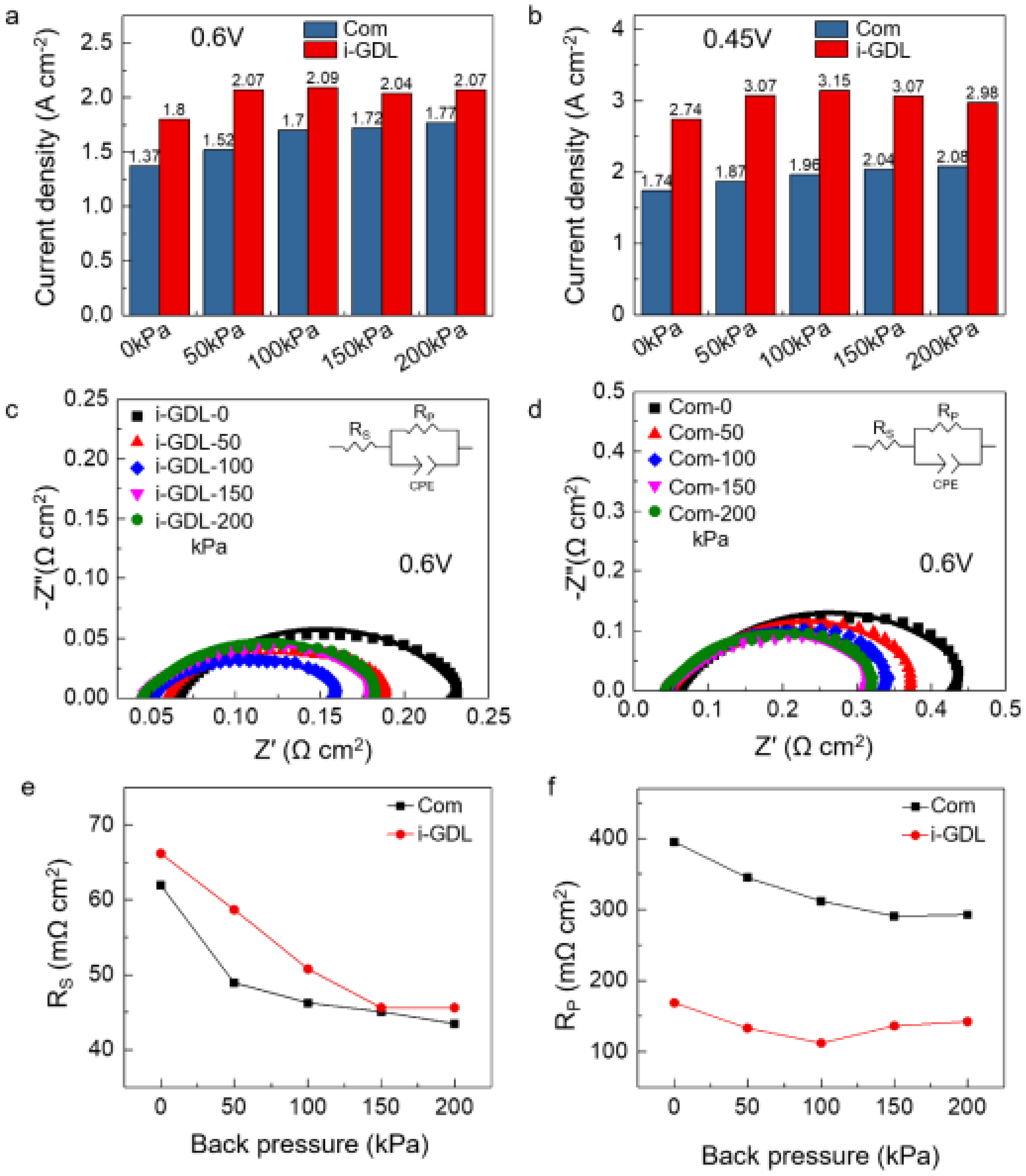

Figure S10. H_2-air PEMFCs performance at 80 °C, 80%/80% anode/cathode humidity, 0 kPa-200 kPa back pressure. (a) Current density of integrated GDL and commercial GDL at 0.6 V at 0 kPa-200 kPa back pressure. (b) Current density of integrated GDL and commercial GDL at 0.45 V at 0 kPa-200 kPa back pressure. (c) The electrochemical impedance spectroscopy of PEMFCs using integrated GDL at 0.6 V at 0 kPa-200 kPa back pressure. (d) The electrochemical impedance spectroscopy of PEMFCs using commercial GDL at 0.6 V at 0 kPa-200 kPa back pressure. (e) The internal resistance for integrated GDL and commercial GDL. (f)The mass transfer resistance for integrated GDL and Commercial GDL.

As shown in **Figure S10**a, the current densities of integrated GDL and commercial GDL are differently pressed at 0.6 V. The current density of commercial GDL gradually increases with the increase of the back pressure. The current density of integrated GDL increases significantly, when the back pressure increases from 0 kPa to 50 kPa. However, with the increasing back pressure, the current density of the integrated GDL does not change significantly.

When the voltage is 0.45 V, the current density of commercial GDL also gradually increases with the gradual increase of the back pressure, as shown in **Figure S10**b. As the back pressure increases from 0 kPa to 100 kPa, the integrated GDL current density increases gradually. When the back pressure is further increased to 200 kPa, the current density decreases slightly. This is because when the back pressure increases to a certain extent, increasing the back pressure does not further improve the mass transfer capacity of the gas, but affects the removal of water, which is also verified from **Figure 3a**.

The EIS were tested at 0.6 V under the conditions of 80 ºC, and 80% RH for both anode and cathode. The gas flow rates of H_2 and air are 0.5 NL min^{-1} and 2.0 NL min^{-1}, respectively. The frequency range of the EIS test was 10 kHz 0.1 Hz. The amplitude of the AC signal was kept at 10% of the DC current. The EIS of the integrated GDL and the commercial GDL as shown in **Figure S10**c-d, the impedance data has been modeled using Zsimpwin software and the $R_s(R_pQ)$ circuit model was used to determine R_s, R_p. In this work, R_s represents the total ohmic resistance of the fuel cell, R_p represents the mass transfer resistance of the fuel cell.[3-8]

Figure S10c shows that the R_s of commercial GDL and integrated GDL gradually decrease with the increase of back pressure. As the back pressure increases, the contact resistance of the fuel cell decreases, and increasing the back pressure can promote the reverse diffusion of water from the cathode to the anode, increasing the proton conductivity, thereby reducing the internal resistance of the fuel cell.

The mass transfer resistance of the commercial GDL and the integrated GDL are shown in **Figure S10**d. When the back pressure increases from 0 kPa to 100 kPa, the gas diffusion of the integrated GDL can be promoted, and the R_p of the integrated GDL decreases from 168 mΩ cm^2 to 112 mΩ cm^2. When the back pressure increases from 100 kPa to 200 kPa, it will affect the discharge of water and affect the gas mass transfer, and R_p will increase from 112 mΩ cm^2 to 142 mΩ cm^2.[9-11] When the back pressure increases from 0 kPa to 150 kPa, the R_p of the commercial GDL decreases from 390 mΩ cm^2 to 290 mΩ cm^2, and when the back pressure increases from 150 kPa to 200 kPa,

the R_p of the commercial GDL remains basically unchanged. By comparison, the R_p of the integrated GDL is much smaller than that of the commercial GDL. The mass transfer resistance of the integrated GDL at 100 kPa is only 0.36 times that of the commercial GDL. In addition, the polarization curves also show that the integrated GDL has almost no concentration polarization, as shown in Figure3a. When the back pressure increases from 0 kPa to 100 kPa, the performance of the fuel cell increases gradually, and when the back pressure increases from 100 kPa to 200 kPa, the performance of the fuel cell decreases slightly. The commercial GDL has a more serious concentration polarization, as shown in Figure3b. When the back pressure increases from 0 kPa to 150 kPa, the performance of the fuel cell increases gradually. When the back pressure increases from 150 kPa to 200 kPa, the performance of the fuel cell exhibits a slight decline. The change trend of polarization curves of integrated GDL and commercial GDL is consistent with that of EIS.

SI-11 Performance under the same configuration of commercial GDL and integrated GDL

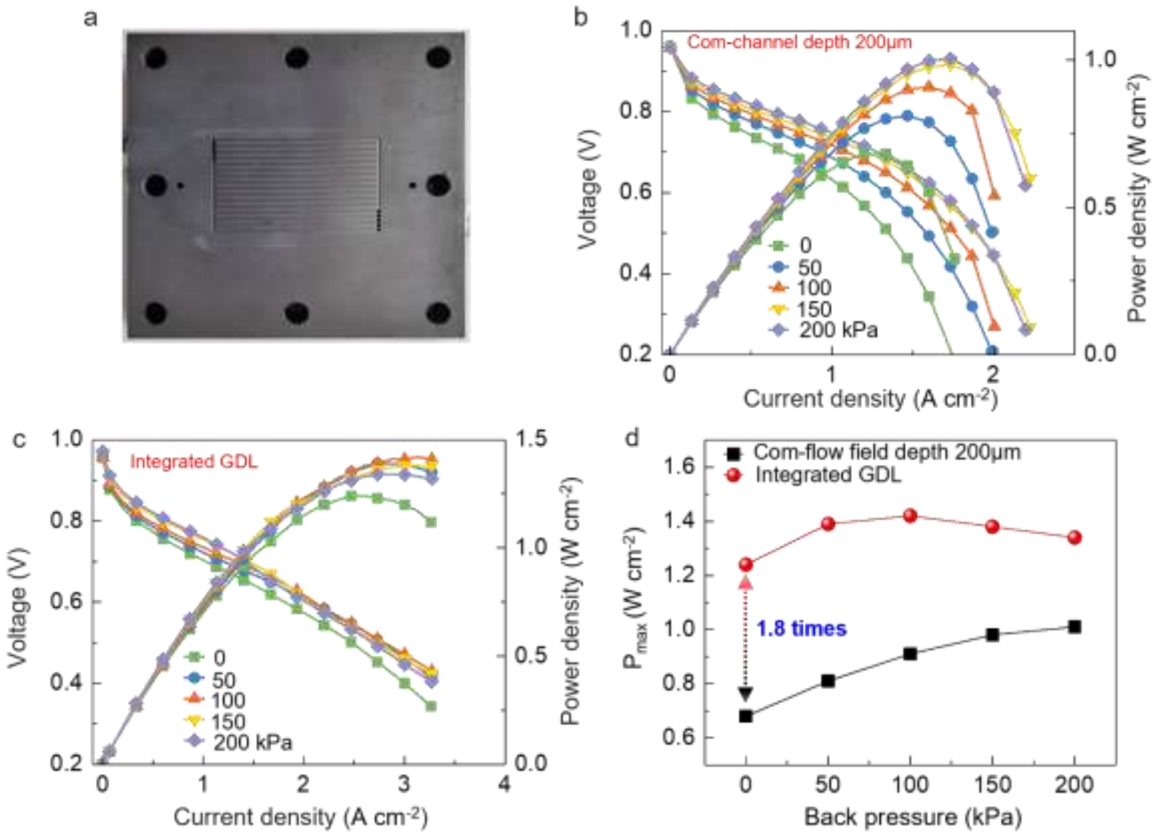

Figure S11. Comparison fuel cell performance of the commercial GDL and the new integrated GDL at the same configuration. (a) Optical photo of traditional flow field plate with the same configuration as the integrated GDL. (b) Polarization curves of the commercial GDL at 0-200 kPa back pressures and 80°C of fuel cell temperature and 80% of humidity. (c) Polarization curves of the new integrated GDL at 0-200 kPa back pressures and 80 °C of fuel cell temperature and 80% of humidity. (d) Comparing the peak power density of the commercial GDL and the new integrated GDL with different back pressures.

In this experiment, the conventional flow field and integrated GDL have the same configuration. The flow field type is parallel flow field, the flow field depth is 200 μm, the flow fields width and ribs width is both 1 mm, as shown in **Figure S11**a.

As shown in **Figure S11**b, it is the fuel cell performance of commercial GDL under 0-200 kPa back pressure. When the back pressure is 0 kPa, the peak power density of commercial GDL is 0.68 W cm^{-2}, and the commercial GDL has a very serious concentration polarization in the concentration polarization region. When the back pressure gradually increases to 200 kPa, the peak power density

of commercial GDL increases from 0.68 W cm^{-2} to 1.01 W cm^{-2}, but there is still serious mass transfer resistance.

When the back pressure is 0 kPa, the peak power density of integrated GDL is 1.24 W cm^{-2}, and the integrated GDL has almost no concentration polarization in the concentration polarization region. When the back pressure increases from 50 kPa to 200 kPa, the integrated GDL fuel cell performance is almost the same, and the power density exceeds 1.3 W cm^{-2}, as shown in **Figure S11**c. Moreover, the peak power density of the integrated GDL is 1.8 times higher than that of the commercial GDL at 0 kPa, as shown in **Figure S11**d.

The flow field parameters of the integrated GDL are the same those of the commercial GDL, which further shows that the integrated GDL wave channels and micro-tunnel ribs can greatly improve the mass transfer capacity, and improving fuel cell performance.

SI-12 Performance of integrated GDL and commercial GDL under pure oxygen conditions

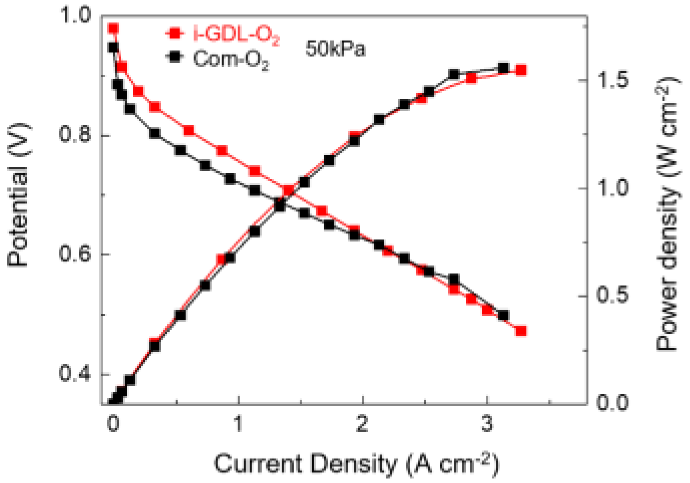

Figure S12. Performance of integrated GDL and commercial GDL under hydrogen-oxygen conditions.

As shown in **Figure S12**, under pure oxygen conditions, the performance of integrated GDLs and commercial GDLs is almost the same.

SI-13 Relationship between air compressor power consumption and back pressure Compressor power

Air compressor is an important part of fuel cell system, in this work, according to the power consumption of the air compressor at different speeds and the corresponding air flow rate at different pressures, we have speculated on the power consumption of the air compressor.

First of all, according to the research of Hu et al.[12-14], we can find that the relationship between the output pressure of the air compressor and the mass flow rate basically conforms to a linear function, which is

$$V_m = -a * P + b \quad (1)$$

Where V_m and P represent air mass flow rate and pressure , a and b are dimensionless constants.

The air volume flow rate is

$$V_v = \frac{V_m}{\rho} = -0.775 * a * P - 0.775 * b \quad (2)$$

Where V_v and ρ represent air volume flow rate and air density.

According to qin research,[12] the relationship between air compressor power consumption and output pressure and gas flow rate is

$$W = -\frac{1.29}{a} V_v + \frac{b}{a} = c * P + d$$
$$= 6.52 * P - 5.51 \quad (3)$$

Where W represent air compressor power consumption.

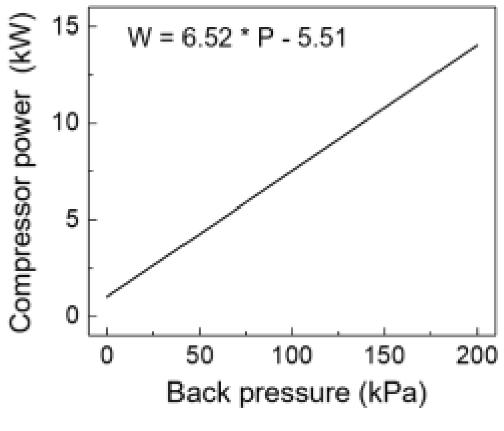

Figure S13. Air compressor power consumption under different back pressure

SI-14 Integrated GDL range prediction

The fuel cell system components are more complex and different components have different power consumption to the system. We refer to the reported work to compare the power consumption percentage of different parts of the fuel cell system[13], the power consumption of the three parts of commercial GDL is shown in **Figure S14**a. Moreover, commercial GDL can achieve better properties only at 200 kPa, while integrated GDL can achieve better fuel cell performance at 50 kPa. The power consumption of the air compressor at 50 kPa is 0.3 times of that at 200 kPa, so the power consumption of the three parts of the integrated GDL is shown in **Figure S14**b. In addition, the integrated GDL range is predicted based on the parameters of the fuel cell vehicle given by Toyota[15].

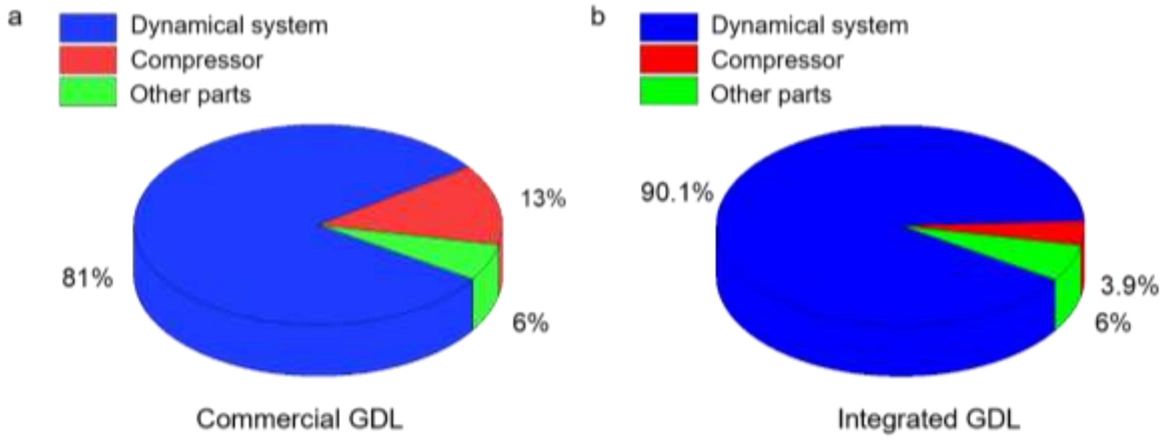

Figure S14. Power consumption ratio of different parts of fuel cell system

Fuel cell system power consumption can be divided into three components, fuel cell output power is consumed by these three components. The details are

$$P = P_D + P_C + P_O \tag{4}$$

Where P, P_D, P_C and P_O represent the total output power, the dynamical system power consumption, the compressor power consumption and the other parts power consumption.

According to the fuel cell parameters given by Toyota, the output power of commercial GDL is 125 kW, according to the calculation of consumption ratio, the power consumption of air compressor is 16.64 kW, and the consumption of other components is 7.68 kW, the actual power system consumption is 103.68 kW, which corresponds to a driving range of 575 km. However, the integrated GDL has a lower compressor power consumption of 4.99 kW compared to the

commercial GDL, so the available power for the powertrain increases to 115.33 kW, corresponding to a driving range of 640 km.

SI-15 Performance of integrated GDLs with different humidity

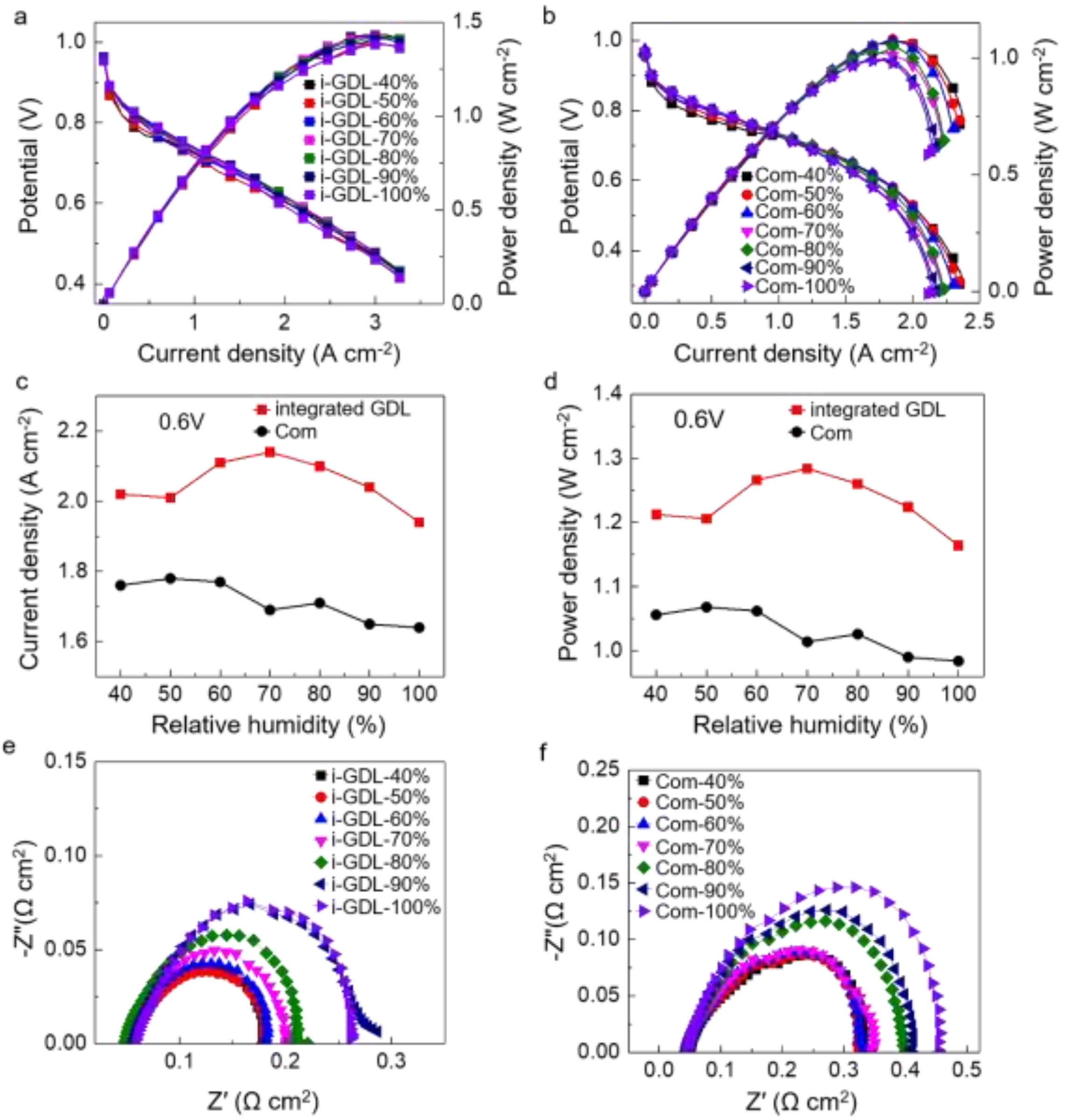

Figure S15. H_2-air PEMFCs performance at 80 °C, 100 kPa back pressure, 40%-100%/40%-100% anode/cathode humidity. (a) The polarization curves of PEMFCs by using integrated GDL at 40%-100% humidity. (b) The polarization curves of PEMFCs by using commercial GDL at 40%-100% humidity. (c) The relationship of integrated GDL and commercial GDL current densities at 0.6 V at 40%-100% humidity. (d) The relationship of integrated GDL and commercial GDL power density at 0.6 V at 40%-100% humidity. (e) The electrochemical impedance spectroscopy (EIS) of PEMFCs

using integrated GDL at 0.6 V at 40%-100% humidity. (f) The electrochemical impedance spectroscopy (EIS) of PEMFCs using commercial GDL at 0.6 V at 40%-100% humidity.

As shown in **Figure S15**a-b, the polarization curve of the integrated GDL did not change significantly when the humidity increased from 40% to 100%. However, the polarization curve of commercial GDL gradually decreases when the humidity increases from 40% to 100%

As shown in **Figure S15**c-d, under the same conditions, the peak power density and current density variations of commercial GDLs and integrated GDLs are consistent. When the voltage is 0.6V and the humidity increases from 40% to 100%, the current density of commercial GDLs decreases gradually. However, the integrated GDL current density first increases gradually and then decreases gradually when the humidity increases from 40% to 100% at 0.6 V. Commercial GDLs have poor water delivery capabilities, and fuel cell performance gradually decreases as humidity increases. The integrated GDL has strong gas mass transfer ability and water management ability, and the proton exchange membrane dries out at low humidity, resulting in low fuel cell performance. As the humidity increases, the proton exchange membrane is gradually wetted, and the fuel cell performance gradually improved. But when the humidity gradually increases to 100, water discharge dominates, resulting in a slight decrease in fuel cell performance.[4, 16-26]

In addition, with the gradual increase of humidity, the electrochemical impedance spectroscopy of integrated GDL and commercial GDL gradual increase (**Figure S15**e-f). But the integrated GDL is 1.5 times less than the commercial GDL.

SI-16 Fuel cells water production calculation

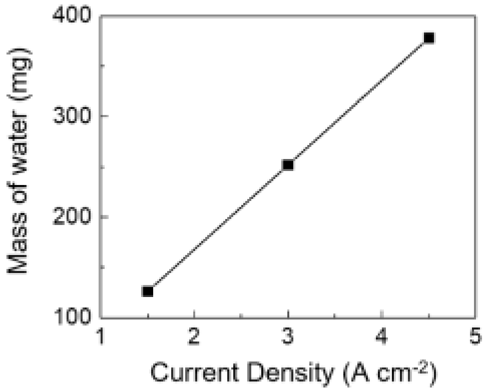

Figure S16. The relationship between current and water volume.

In this work, we calculate the water production in one minute according to the standard of 1.5 A cm^{-2}, 3 A cm^{-2}, and 4.5 A cm^{-2}, and MEA area is 15 cm^2. The production of water is due to the occurrence of oxygen reduction reaction[27] (ORR), and the (ORR) on the cathode side is

$$\frac{1}{2}O_2 + 2H^+ + 2e^- \rightarrow H_2O \tag{5}$$

The amount of charge generated by electrons in one minute

$$Q = i * 15 * t \tag{6}$$

Where Q represent quantity of electric charge, i represent current density, t represent time.

The moles of electrons is

$$n(e^-) = \frac{Q}{F} \tag{7}$$

Where $n(e^-)$ represent moles of electrons, F represent the Faraday's constant.

The moles of water is

$$n(water) = \frac{1}{2}\, n(e^-) \tag{8}$$

Where $n(water)$ represent moles of water.

The mass of water is

$$m(water) = \frac{n(water)}{M(water)} \tag{9}$$

Where $m(water)$ represent mass of water, $M(water)$ represent the molar mass of water.

SI-17 The removal time of different water volumes (the stoichiometric ratio of air flow is 2)

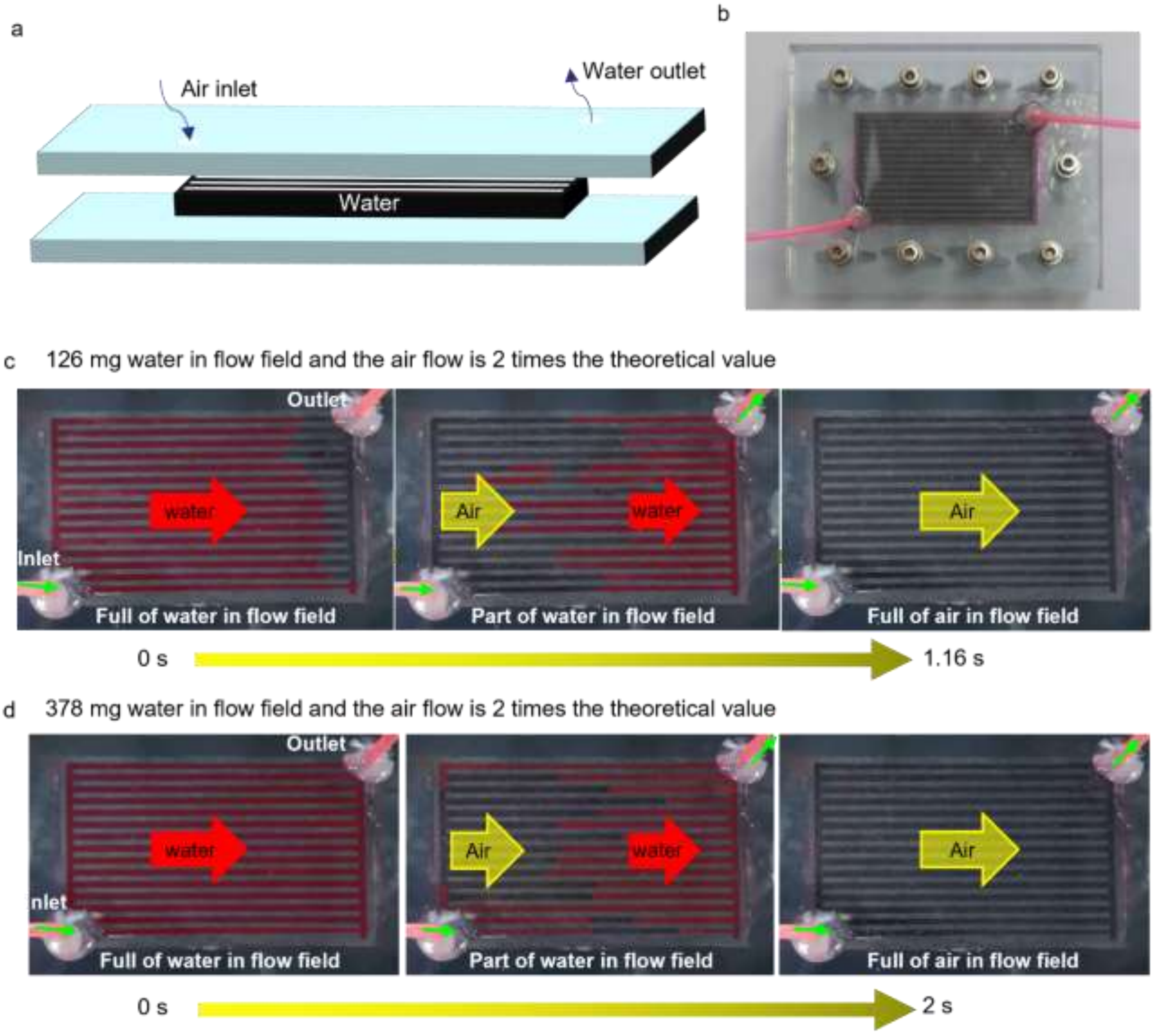

Figure S17. Liquid water discharge in the flow channel when the air flow rate is twice of the theoretical air flow rate. (a) Schematic diagram of the integrated GDL drainage test setup. (b) Integrated GDL water discharge test device. (c) The discharge time of water in the flow field when the water in the flow field is 126 mg. (d) The discharge time of water in the flow field when the water in the flow field is 378 mg.

The test device for the drainage experimental is shown in **Figure S17**a-b. Inject water produced under different current densities within 1 minute into the flow channel, pass a certain flow of gas,

and record the time from the gas entering to the liquid being completely eliminated.

When the current density is 1.5 A cm^{-2}, the water generated in one minute is injected into the flow channel, and the amount of gas twice as much as in theory is injected. The test shows that it only takes 1.16 seconds to discharge all the water, as shown in **Figure S17**c. When the current density is 4.5 A, the water generated in one minute is injected into the flow channel, and the gas with the same flow rate is injected, and it takes 2 seconds for all the water to be discharged, as shown in **Figure S17**d.

SI-18 The removal time of different water volumes (the stoichiometric ratio of air flow is 1.5)

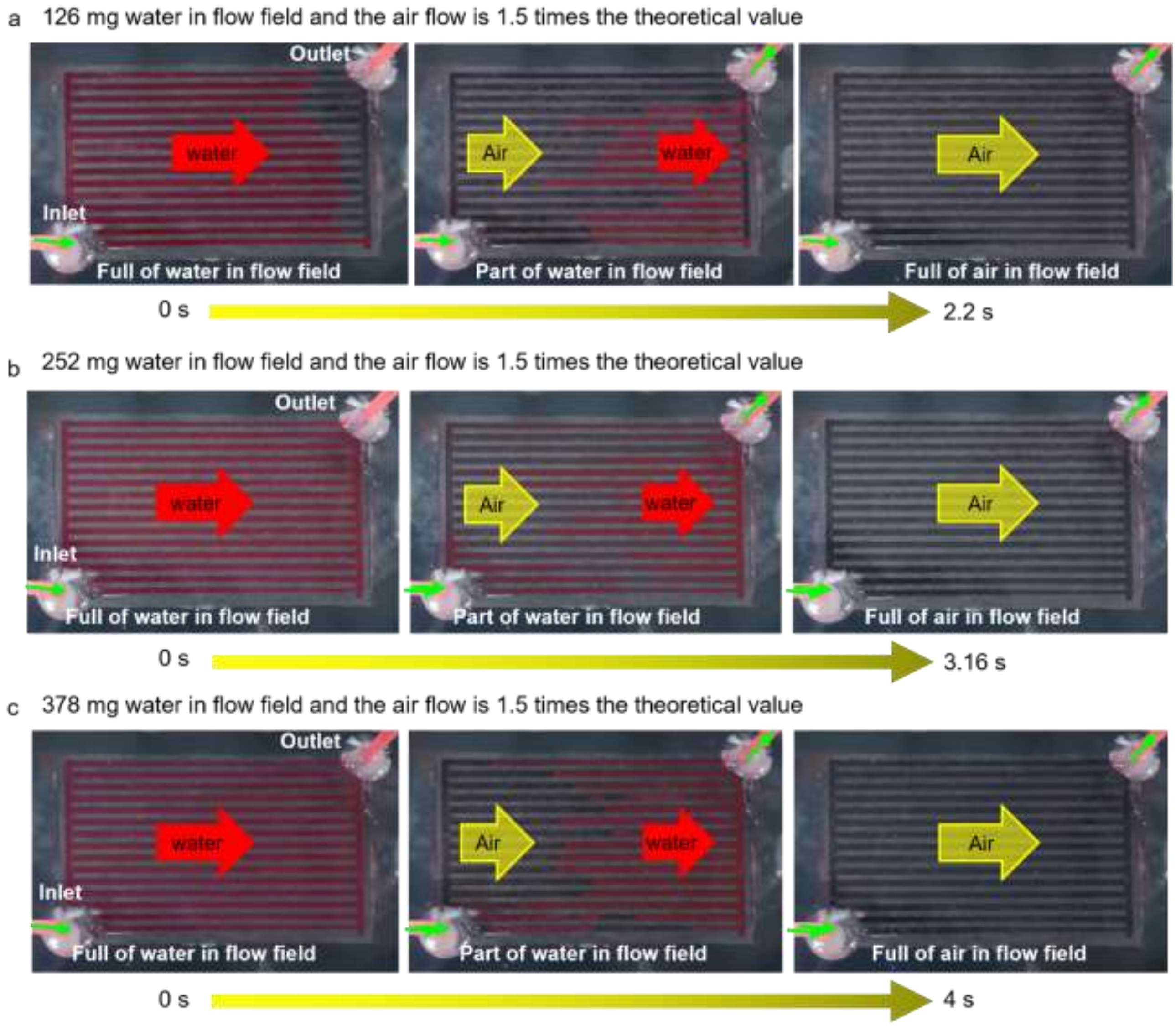

Figure S18. The discharge of liquid water in the flow channel when the air flow rate is 1.5 times of the theoretical air flow rate. (a) The discharge time of water in the flow field when the water in the flow field is 126 mg (b) The discharge time of water in the flow field when the water in the flow field is 252 mg (c) The discharge time of water in the flow field when the water in the flow field is 378 mg.

When the current density is 1.5 A cm^{-2}, the water generated in one minute is injected into the flow channel, and the gas that is 1.5 times the theoretical gas flow is introduced. The test shows that it takes 2.2 seconds to discharge all the water, as shown in **Figure S18**a. When the current density increases to 3 A cm^{-2}, the amount of water produced in one minute is 252 mg, and the gas with the

same flow rate is injected. The test shows that it takes 3.16 seconds to discharge all the water, as shown in **Figure S18**b. When the current density increases to 4.5 A cm^{-2}, the water generated in one minute is injected into the flow channel, and the gas that is 1.5 times the theoretical gas flow is introduced. The test shows that it takes 4 seconds to discharge all the water, as shown in **Figure S18**c.

SI-19 Integrated GDL gaseous water testing device

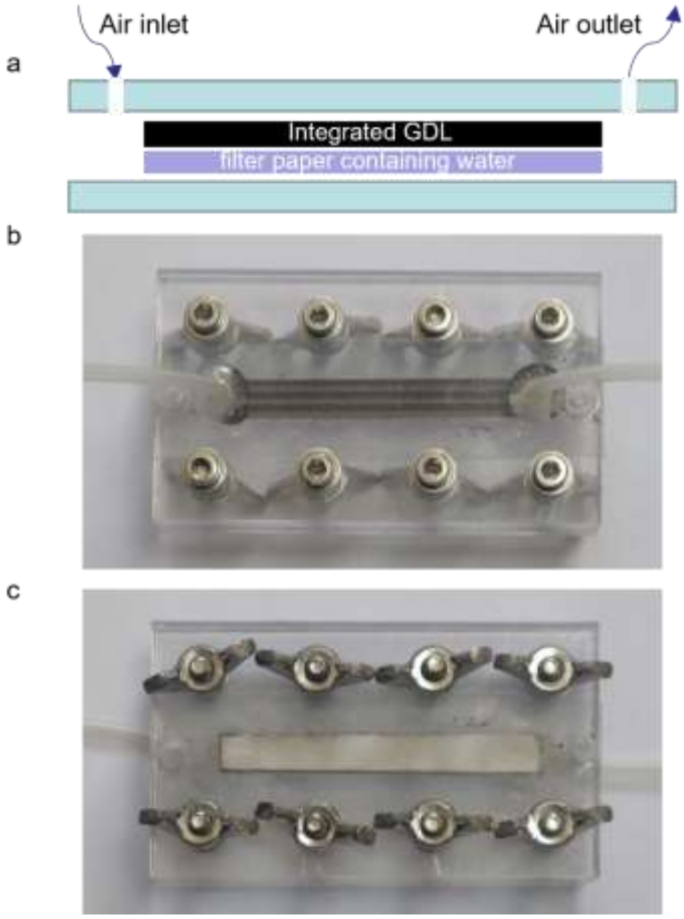

Figure S19. Integrated GDL visual test device. (a) Schematic diagram of integrated GDL test device. (b) Top view of GDL gas water emission test device. (b) Upside down view of GDL gas water emission test device.

SI-20 Comparison of gas water transfer for different GDLs

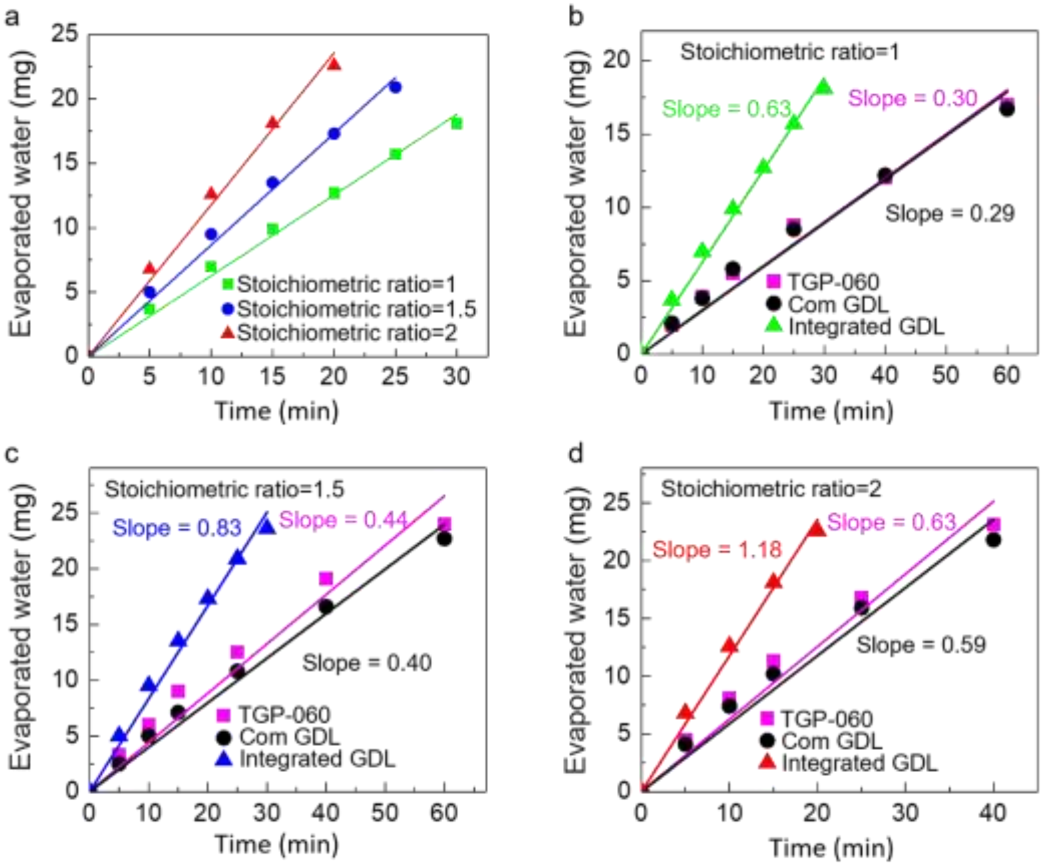

Figure S20. Traditional flow field and integrated GDL water loss rates. (a) The water loss rate of the integrated GDL when the air flow is 1 time, 1.5 times and 2 times the theoretical values of integrated GDL. (b) The water loss rate of the integrated GDL, TGP-060 and commercial GDL when the air flow is 1 time the theoretical value. (c) The water loss rate of the integrated GDL, TGP-060 and commercial GDL when the air flow is 1.5 times the theoretical values. (d) The water loss rate of the integrated GDL, TGP-060 and commercial GDL when the air flow is 2 times the theoretical values.

We performed gaseous water discharge tests using the setup shown in **Figure S20**. In the fuel cells composed of integrated GDL, the larger the gas flow rate, the faster the gaseous water discharge rate, as shown in **Figure S20**a.

We added the water evaporation experiment of TGP-060 to compare with the integrated GDL, as shown in **Figure S20**. The thickness of TGP-060 is basically the same as that of the integrated GDL bottom, and both have the same PTFE content. At the same time, the traditional flow field parameters used by TGP-060 are consistent with the integrated GDL flow field parameters. We placed filter paper containing 25 mg of water at the bottom of the GDL, passed the corresponding

airflow through the system, and recorded the mass every 5-10 minutes until the mass stopped changing. Under the same mass reduction, the GDL gaseous water that takes a short time is discharged quickly.

When the metering ratio is 1, the water evaporation rate of TGP-060 is 0.3 mg/(min cm^2). When the metering ratio is 1.5, the water evaporation rate of TGP-060 is 0.44 mg/(min cm^2). When the metering ratio is 2, the water evaporation rate of TGP-060 is 0.63 mg/(min cm^2), as shown in **Figure S20**b-d. Under different gas stoichiometric ratios, TGP-060 and commercial GDL gaseous water discharge rate is almost the same, and the water evaporation rate of the integrated GDL is almost 2 times that of the TGP-060.

The results showed that the integrated GDL had a higher water evaporation rate than TGP-060 and commercial GDL under the same conditions. This also proves that the integrated GDL has an excellent water evaporation rate due to the wave channels and micro-tunnel ribs, rather than PTFE content and thickness.

SI-21 Performance comparison of integrated GDL and TGP-060

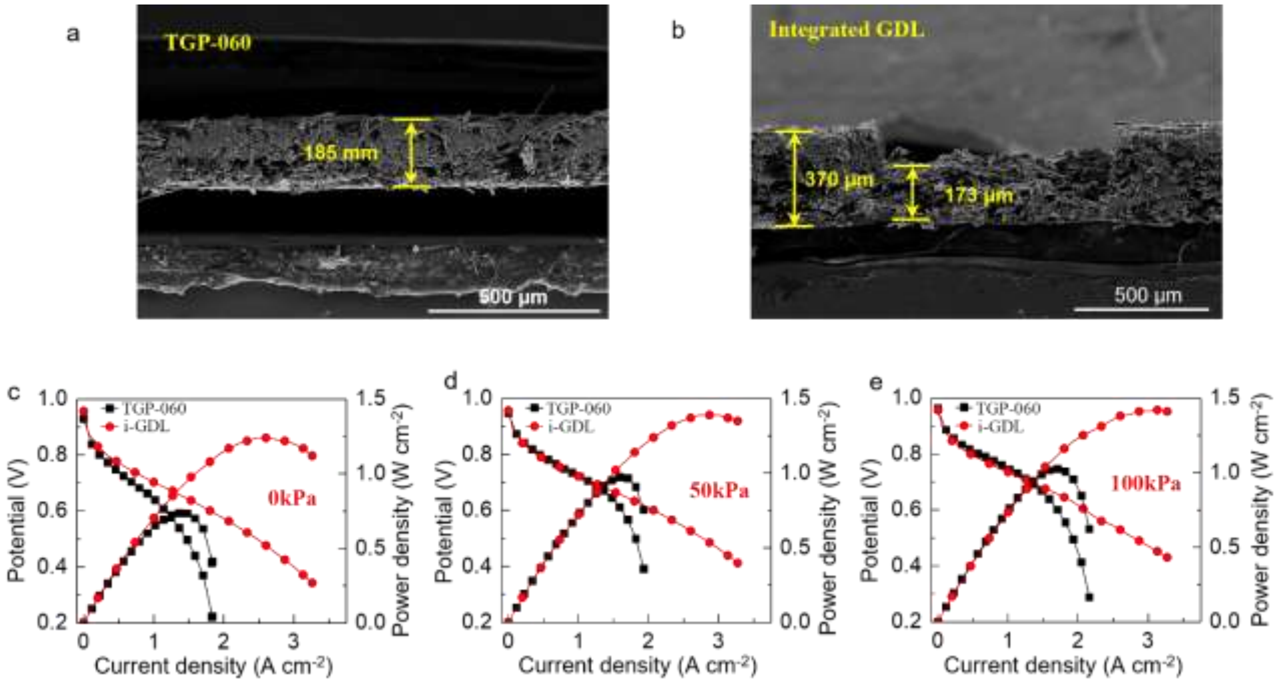

Figure S21. Comparison fuel cell performance of the TGP-060 and the new integrated GDL. PEMFCs polarization curves were recorded under different back pressures and 80 °C of fuel cell temperature and 80% of relative humidity, the gas flow rates of H_2 and air are 0.5 NL min^{-1} and 2 NL min^{-1}, respectively. (a) Cross section SEM image of the TGP-060. (b) Cross section SEM image of the new integrated GDL. (c) Polarization curves of the TGP-060 and the new integrated GDL at 0 kPa back pressures. (d) Polarization curves of the TGP-060 and the new integrated GDL at 50 kPa back pressures. (e) Polarization curves of the TGP-060 and the new integrated GDL at 100 kPa back pressures.

In order to further illustrate that the excellent fuel cell performance is due to the micro-tunnel ribs and wave channels of our new integrated GDL, not the thinner thickness of the integrated GDL. We used TGP-060 (by Toray) as the GDL, and compare the fuel cell performance with the integrated GDL, as shown in **Figure S21**. The thickness of TGP-060 is 186 μm, as shown in **Figure S21**a. The thickness of the bottom of our integrated GDL is 173 μm, as shown in **Figure S21**b. TGP-060 is basically the same thickness as the bottom layer of integrated GDL, and the other test conditions are exactly the same.

The results show that the integrated GDL has excellent fuel cell performance compared with TGP-060 at 0-100 kPa, as shown in **Figure S21**c-e. With the increase of back pressure, although the

fuel cell performance of TGP-060 under the traditional flow channel has been improved to a certain extent, but the concentration polarization area has very serious concentration polarization. By comparison, the integrated GDL exhibits better mass transfer capability in the concentration-polarized region with and without backpressure. The reason is that the integrated GDL has a special structure of wave channels and micro-tunneled ribs, which leads to better fuel cell performance of the integrated GDL.

SI-22 The flow form of gas in the flow channel

The Reynolds number is a dimensionless constant that characterizes fluid flow. A Reynolds number less than 2300 indicates laminar flow, while a Reynolds number between 2300 and 4000 signifies a transitional state, and a Reynolds number greater than 4000 denotes turbulent flow. Calculating the Reynolds number allows us to estimate the fluid flow state in the channel.

In fuel cell testing, the gas is quantified by its volumetric flow rate, which must be converted to a flow rate to calculate the Reynolds number. The conversion formula of flow rate and flow rate is

$$V = \frac{Q}{\pi \times (\frac{d}{2})^2} \tag{10}$$

Where V represents gas flow rate, Q represents the volumetric flow rate, and d represents pipe diameter.

The formula for calculating the Reynolds number is

$$Re = \frac{\rho V d}{\mu} \tag{11}$$

Where Re represents Reynolds number, V represents gas flow rate, d represents pipe diameter, and μ represents fluid viscosity coefficient (When the temperature is 15 degrees Celsius and the atmospheric pressure is on 101325 Pascals, μ is equal to 1.81×10^{-5}, and the unit is Pa · s).

The gas flow inside a fuel cell's flow field exhibits different states at various positions, leading us to treat the flow path as an ideal pipe for computation. When the gas flow rate is 2000 mL min^{-1}, The traditional flow field with a 1 mm channel diameter exhibits a Reynolds number of 3020, whereas the integrated gas diffusion layer (GDL) with a channel diameter of 200 μm results in a Reynolds number of 15100 These Reynolds numbers reveal that the gas flow in the traditional flow

field channel is transitional, whereas the integrated GDL channel exhibits turbulent flow.

SI-23 Summary of volume specific power density of fuel cells literatures in recent year

Table S1. Summary of volume specific power density of fuel cells in the literatures.

Number	Year	Volume specific power density (W L^{-1})	Reference
1	1998	300	51
2	2004	500	50
3	2006	360	52
4	2008	3107	53
5	2010	3040.9	54
6	2010	2715.94	55
7	2012	5764	56
8	2016	3100	58
9	2017	5190	57
10	2022	16322	This work

References

[1] S. G. Goebel, *J. Power Sources* **2011**, *196*, 7550.

[2] D. H. Jeon, *J. Power Sources* **2019**, *423*, 280.

[3] A. Kulikovsky, *J. Electrochem. Soc.* **2019**, *166*, F306.

[4] E. Janicka, M. Mielniczek, L. Gawel, K. Darowicki, P. Landowska, *Electrochim. Acta* **2020**, *341*.

[5] A. V. Shirsath, S. Raël, C. Bonnet, L. Schiffer, W. Bessler, F. Lapicque, *Curr Opin Electrochem* **2020**, *20*, 82.

[6] T. Ma, W. Lin, Z. Zhang, J. Kang, Y. Yang, *Int. J. Hydrog. Energy* **2021**, *46*, 17388.

[7] H. Yuan, H. Dai, X. Wei, P. Ming, *Chem. Eng. J.* **2021**, *418*.

[8] X. Zhu, L. Su, X. Wang, R. Chen, D. Ji, Y. Ma, L. Wu, J. Zhang, W. Zhou, *Energy Convers. Manag.* **2023**, *281*, 116856.

[9] C. Cai, Y. Rao, Y. Zhang, F. Wu, S. Li, M. Pan, *Int. J. Hydrog. Energy* **2019**, *44*, 13786.

[10] J. Zhang, H. Li, J. Zhang, *ECS Trans* **2009**, *19*, 65.

[11] D. Ko, S. Doh, H. S. Park, M. H. Kim, *Renew. Energ.* **2018**, *115*, 896.

[12] Y. Qin, Q. Du, M. Fan, Y. Chang, Y. Yin, *Energy Convers. Manag.* **2017**, *142*, 357.

[13] B. Zhang, X. Wang, D. Gong, S. Xu, *Int. J. Hydrog. Energy* **2022**, *47*, 21417.

[14] D. Hu, J. Liu, F. Yi, Q. Yang, J. Zhou, *Energy Convers. Manag.* **2022**, *251*, 115007.

[15] TOYATA, *2022 Toyota Mirai Specs & Options*, https://www.toyota.com/mirai/2022/features/mpg_other_price/3002/3003/, 3, **2022**.

[16] B. Cheng, O. Minggao, Y. Baolian, *Tsinghua Sci Technol* **2006**, *11*.

[17] M. M. Saleh, T. Okajima, M. Hayase, F. Kitamura, T. Ohsaka, *J. Power Sources* **2007**, *164*, 503.

[18] X.-D. Wang, Y.-Y. Duan, W.-M. Yan, F.-B. Weng, *J. Power Sources* **2008**, *176*, 247.

[19] P. H. Lee, S. S. Hwang, *Sensors* **2009**, *9*, 9104.

[20] D. H. Jeon, K. N. Kim, S. M. Baek, J. H. Nam, *Int. J. Hydrog. Energy* **2011**, *36*, 12499.

[21] P. K. Takalloo, E. S. Nia, M. Ghazikhani, *Energy Convers. Manag.* **2016**, *114*, 290.

[22] Q. Zhang, R. Lin, L. Técher, X. Cui, *Energy* **2016**, *115*, 550.

[23] T. Wilberforce, O. Ijaodola, F. N. Khatib, E. O. Ogungbemi, Z. El Hassan, J. Thompson, A. G. Olabi, *Sci. Total Environ.* **2019**, *688*, 1016.

[24] M. Baghban Yousefkhani, H. Ghadamian, K. Daneshvar, N. Alizadeh, B. C. Rincon Troconis, *Energies* **2020**, *13*, 6117.

[25] Z. Cheng, L. Luo, B. Huang, Q. Jian, *Int. J. Hydrog. Energy* **2021**, *46*, 26560.

[26] J. Zhao, Z. Tu, S. H. Chan, *Energy* **2022**, *239*, 122270.

[27] Q. Wen, S. Pan, Y. Li, C. Bai, M. Shen, H. Jin, F. Ning, X. Fu, X. Zhou, *ACS Energy Lett.* **2022**, *7*, 3900.
